# Metabolic Glycan Labeling of Cancer Cells Using Variably Acetylated Monosaccharides

**DOI:** 10.1021/acs.bioconjchem.2c00169

**Published:** 2022-07-25

**Authors:** Daniel R. Parle, Flaviu Bulat, Shahd Fouad, Heather Zecchini, Kevin M. Brindle, André A. Neves, Finian J. Leeper

**Affiliations:** † Yusuf Hamied Department of Chemistry, University of Cambridge, Lensfield Road, Cambridge CB2 1EW, United Kingdom; ‡ Cancer Research UK Cambridge Institute, University of Cambridge, Li Ka Shing Centre, Robinson Way, Cambridge CB2 0RE, United Kingdom

## Abstract

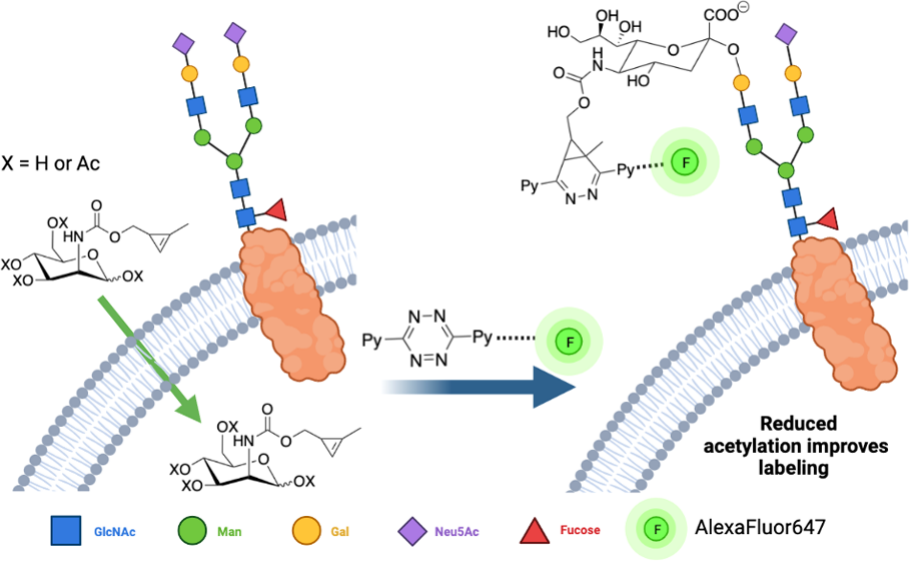

Methylcyclopropene (Cyoc)-tagged tetra-acetylated monosaccharides, and in particular mannosamine derivatives, are promising tools for medical imaging of cancer using metabolic oligosaccharide engineering and the extremely fast inverse electron-demand Diels–Alder bioorthogonal reaction. However, the *in vivo* potential of these monosaccharide derivatives has yet to be fully explored due to their low aqueous solubility. To address this issue, we sought to vary the extent of acetylation of Cyoc-tagged monosaccharides and probe its effect on the extent of glycan labeling in various cancer cell lines. We demonstrate that, in the case of Ac*_x_*ManNCyoc, tri- and diacetylated derivatives generated significantly enhanced cell labeling compared to the tetra-acetylated monosaccharide. In contrast, for the more readily soluble azide-tagged sugars, a decrease in acetylation led to decreased glycan labeling. Ac_3_ManNCyoc gave better labeling than the azido-tagged Ac_4_ManNAz and has significant potential for *in vitro* and *in vivo* imaging of glycosylated cancer biomarkers.

## Introduction

Abnormal glycosylation has been associated with a variety of diseases, including cancer,^1,2^ highlighting the vital role glycans play in many cellular interactions. This aberrant glycosylation is involved in tumor proliferation and progression, angiogenesis, invasion, metastasis, and immunomodulation.^3,4^ Altered cancer cell-surface glycoproteins are a target for molecular imaging and targeted therapeutics.^5−7^

Abnormal glycosylation is often the result of changes in the monosaccharide composition of the glycans. These include increased *N*-glycan branching via GlcNAc,^8^ an increase in mucin-type glycans initiated by GalNAc,^6,9^ and increased sialylation (synthesized intracellularly from ManNAc).^10−12^ Consequently, these glycan signatures offer potential diagnostic and therapeutic targets for exploiting the aberrant glycan structures displayed by tumor cells.

Metabolic oligosaccharide engineering (MOE) has been widely used as a technique for labeling glycans in several tissue types.^13^ A variety of bioorthogonal reactions have been exploited to achieve this, including Staudinger ligations,^14^ inverse electron-demand Diels–Alder (IED-DA) reactions,^15^ and azide-alkyne cycloadditions.^16^ For metabolic labeling, the nature of the chemical reporter on the monosaccharide is key. Large motifs are often not tolerated by glycosyltransferase enzymes in the biosynthetic pathways of glycan production; small chemical reporters such as azide^14^ and isonitrile^17^ groups are generally thought to be preferred.

IED-DA reactions can show very fast kinetics for bioorthogonal ligation reactions and can be orthogonal to the widely used strain-promoted alkyne-azide cycloaddition (SPAAC) reactions, allowing both reactions to be used concurrently in dual-labeling studies.^18−20^ While many motifs such as *trans*-cyclooctenes show very rapid IED-DA kinetics with tetrazines,^15^ their use in metabolic labeling is limited due to their relatively large size, which limits their incorporation. However, the smaller cyclopropene motif is better tolerated by glycosyltransferases for glycan incorporation^21^ and is hence an exciting novel tool for MOE, along with its fast reaction partner, tetrazine (Tz).

**Figure 1:**
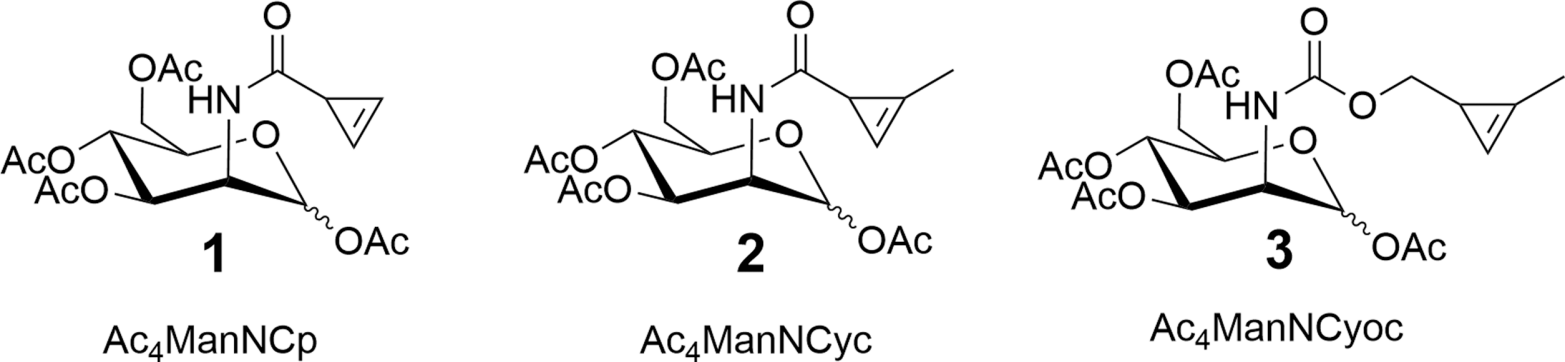
Different cyclopropene derivatives of mannosamine (ManN) that have been reported.

Unsubstituted cycloprop-2-ene-1-carbonyl (Cp) derivatives are generally unstable. Despite this, Ac_4_ManNCp^22^**1** (Figure 1) and other Cp-labeled sugars^23^ have been used for MOE. Methyl cyclopropenes^21,24^ (Cyc) such as ManNCyc (**2**) are more stable and thus suitable for MOE^25^ but reaction speeds with tetrazines are ca. threefold slower than unmethylated analogues.^18^ However, both ManNCp (**1**) and ManNCyc (**2**) groups have a carbonyl group directly attached to the cyclopropene, and this electron-withdrawing group slows down the IED-DA reaction with tetrazines by more than 50-fold relative to carbamate-linked cyclopropene (Cyoc) groups, as in **3**.^26^ The enhanced kinetics of the carbamate compensate for the reduced incorporation of **3** relative to **1** and **2**.^23^ ManNCyoc **3** was shown to react with a tetrazine with a rate constant of 0.99 M^–1^ s^–1^ at 20 °C.^18^ Ac_4_ManNCyoc has, for example, been used to metabolically label a human leukemic T-lymphocyte line (Jurkat) and its incorporation into cell-surface glycans was imaged with either a Tz-biotin/avidin combination or a directly linked Tz-fluorophore,^26^ targeting the increased levels of sialylation observed in cancer cell lines.

**Figure 2:**
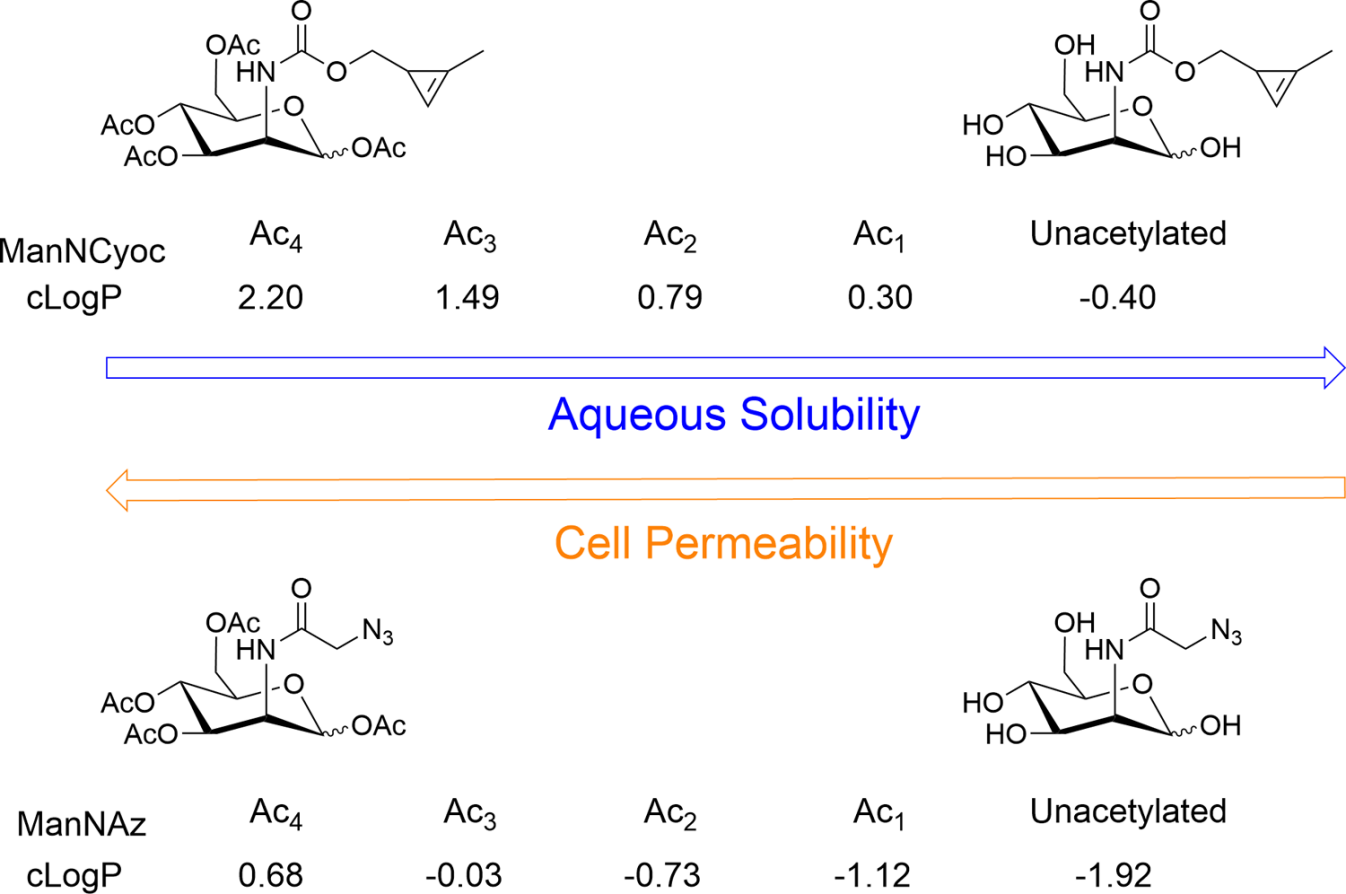
Striking a balance between aqueous solubility and cell permeability by varying the degree of acetylation of Cyoc- and azido-tagged monosaccharides. The *c* log *P* values of Ac*_x_*ManNCyoc and Ac*_x_*ManNAz are shown as examples.^31^

Tetra-acetylated monosaccharides are generally used for MOE to aid cell permeability, as the unacetylated monosaccharides are too polar for passive diffusion through the plasma membrane (Figure 2). After cell uptake, the acetylated monosaccharides are deacetylated by nonspecific esterases in the cytosol.^27^ Despite demonstrations of *in vitro* ligation reactions, *in vivo* applications of cyclopropene-tagged sugars have been limited, to our knowledge, to a single report^22^ using **1** with detection of the label performed *ex vivo*. This may be a consequence of the poor aqueous solubility of Ac_4_ManNCyoc, even in the presence of a cosolvent (e.g. 10% DMSO). In contrast, tetraacetylated azido-tagged sugars have been widely used for *in vivo* applications.^28−30^ We believe this difference is due to the better solubility of the azido-tagged sugars relative to their cyclopropene counterparts, which is related to their reduced *c* log *P* (Figure 2).

Here, we investigate whether partially acetylated Cyoc-tagged sugars are more effective for MOE than the peracetylated versions. We show that the di- and triacetylated ManNCyoc derivatives are not only more soluble in aqueous media than Ac_4_ManNCyoc but also better incorporated into cell-surface glycans *in vitro* and, therefore, more suitable for use in MOE experiments.

## Results and Discussion

### Synthesis of Monosaccharides

Unacetylated cyclopropene-tagged mannosamine (ManNCyoc), galactosamine (GalNCyoc), and glucosamine (GlcNCyoc) derivatives were prepared using previously reported methods.^26^ The tetraacetylated derivatives were synthesized by reacting the unacetylated tagged sugars with excess pyridine and acetic anhydride, as described previously.^26^ The same approach was used for azido-tagged ManNAz, GalNAz, and GlcNAz.^32^

**Figure 3:**
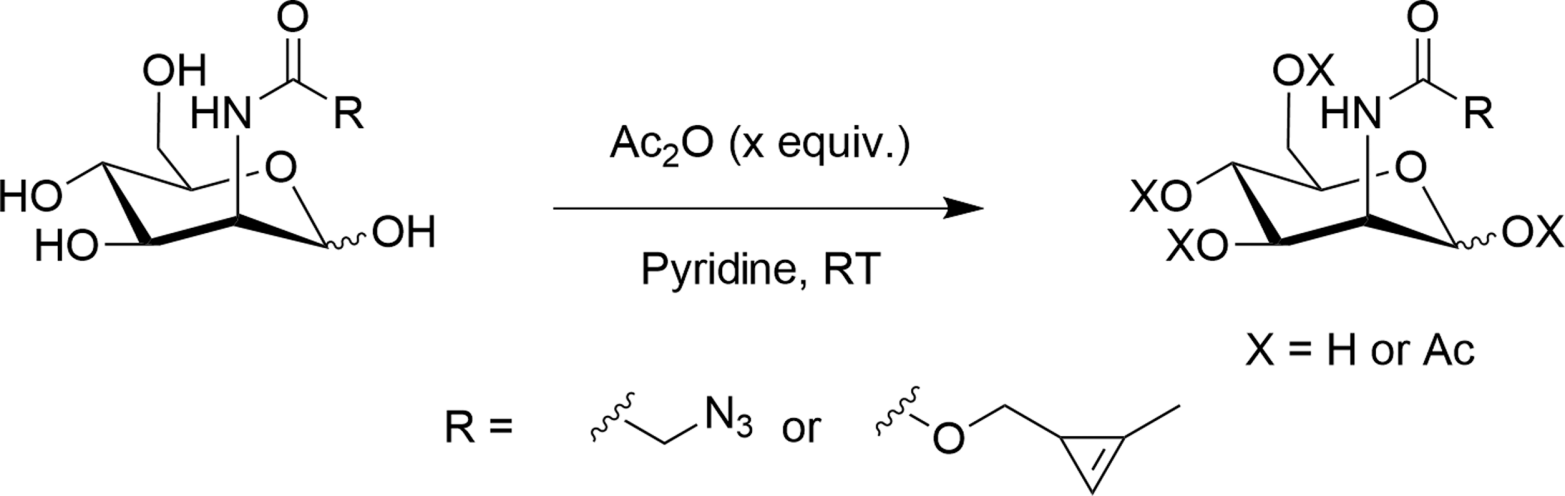
Synthesis of mono- and diacetylated monosaccharides. Acetylation of the ManN derivatives is shown as an example.

While methods exist to synthesize many different selectively acetylated sugar derivatives,^33^ the standard procedures largely use acidic conditions (for acetal hydrolysis), hydrogenation (for removal of benzyl groups), and/or oxidative conditions (e.g., for removal of *p*-methoxybenzyl groups or activation of an anomeric leaving group). Unfortunately, the Cyoc group does not tolerate any of these conditions^22,23^ and even decomposes upon heating to 80 °C. We were, therefore, very limited in the types of reactions we could use. To synthesize mono- and diacetylated Cyoc-tagged derivatives, the unacetylated sugars were dissolved in pyridine and the corresponding stoichiometric amount of acetic anhydride added (Figure 3). This approach, however, did not yield a single level of acetylation but instead a range of different degrees of acetylation as well as different regioisomers, resulting in a complex mixture of products, as detected by NMR and LCMS. However, the more polar products could be partially separated by multiple rounds of normal phase chromatography. This separated the mono- and diacetylated sugars for both the azide and cyclopropene series with good control over the level of acetylation but no control over the position of acetylation.

**Figure 4:**
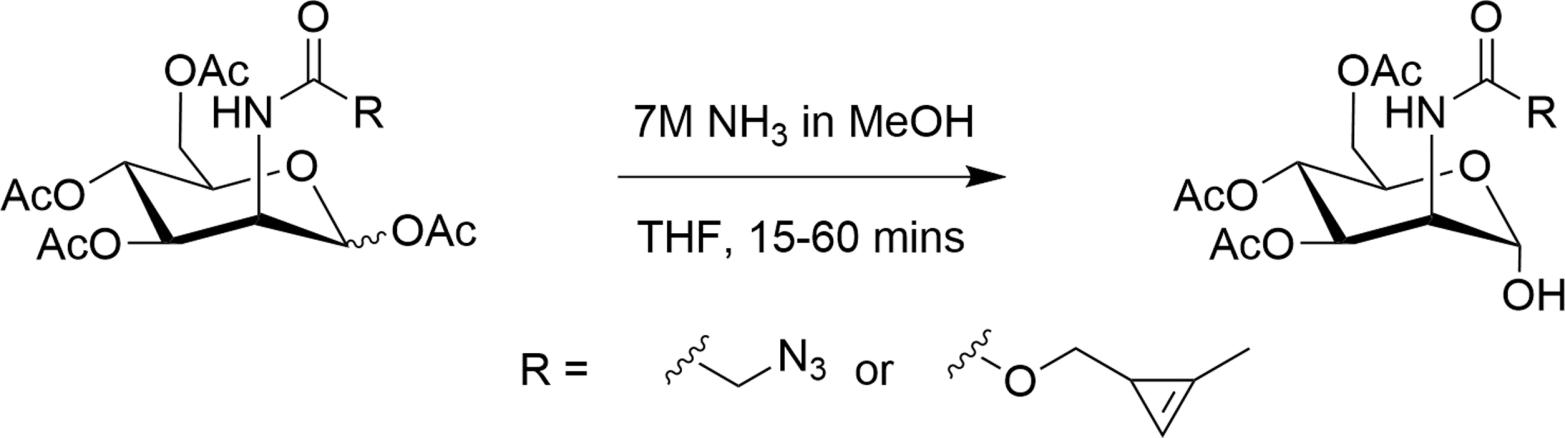
Selective synthesis of triacetylated monosaccharides via anomeric deacetylation. Deacetylation of the Ac_4_ManN derivatives is shown as an example.

For the triacetylated derivatives, we used instead selective deacetylation of the corresponding tetraacetylated monosaccharide. Numerous reagents have been used to achieve selective deacetylation including hydrazine hydrate,^34^ ammonium carbonate,^35^ and zinc acetate.^36^ We found that addition of 7 M ammonia in methanol to the tetra-acetylated sugar dissolved in THF, as described by Fiandor et al.,^37^ resulted in selective conversion to the triacetylated sugar in 2 h and was compatible with both the azide and cyclopropene tags (Figure 4). We confirmed by NMR that it is the anomeric position that is deacetylated (Figure S2.1). This method was used to synthesize pure triacetylated analogues of each tagged sugar in this study, with excellent regioselectivity and anomeric control (α-selective), as described in the initial report of this method.^37^

In summary, we synthesized mono- and diacetylated azido and Cyoc-tagged ManN, GalN, and GlcN with no control over the position of acetylation but good control of the level of acetylation. Tri-acetylated sugars, on the other hand, were synthesized as pure compounds, with respect to the position and level of acetylation and stereochemistry at the anomeric position (further discussed in Section 2.1 of the Supporting Information). In contrast, previous studies with tetra-acetylated Cyoc-tagged sugars have all used a mixture of anomers.

### *In Vitro* Labeling

**Figure 5:**
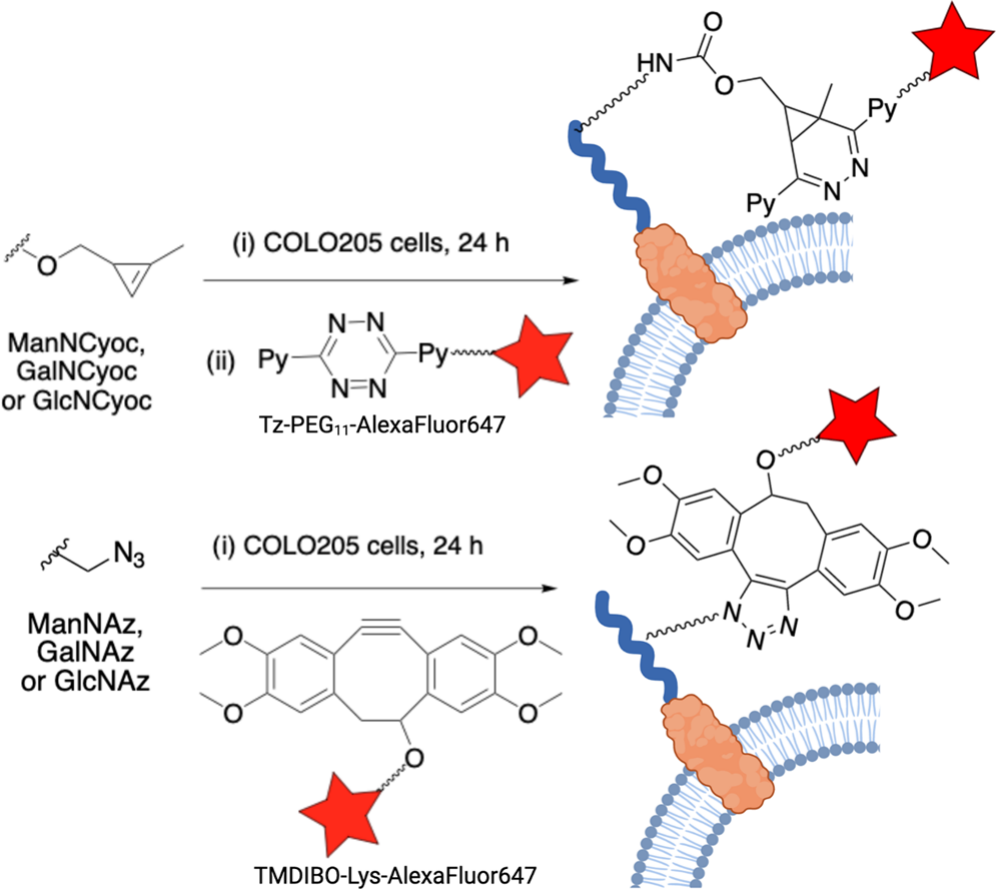
*In vitro* labeling strategy. The figure was created with BioRender.com.

Next, we investigated the effect of the degree of acetylation of these tagged sugars on metabolic labeling in colorectal adenocarcinoma cells (COLO205). COLO205 cells were incubated with 125 μM of each of the differently acetylated Cyoc-tagged sugars: Ac, Ac_2_, Ac_3_, and Ac_4_. Cyoc-tagged sugars were detected using a Tz-PEG_11_-AlexaFluor647 dye^38^ and azido-tagged sugars using a TMDIBO-Lys-AlexaFluor647 dye (Figure 5).^39^

A direct bioorthogonal reaction was used for labeling rather than using a biotinylated probe followed by detection using a fluorescently tagged streptavidin. While the two-step approach should lead to a reduced background signal and hence an increased signal-to-background ratio (SBR),^40^ it would be less applicable *in vivo*. Whereas, by using a fluorescently labeled tetrazine, this same system could then be directly translated to an experiment *in vivo*, using a single bioorthogonal reaction.

**Figure 6:**
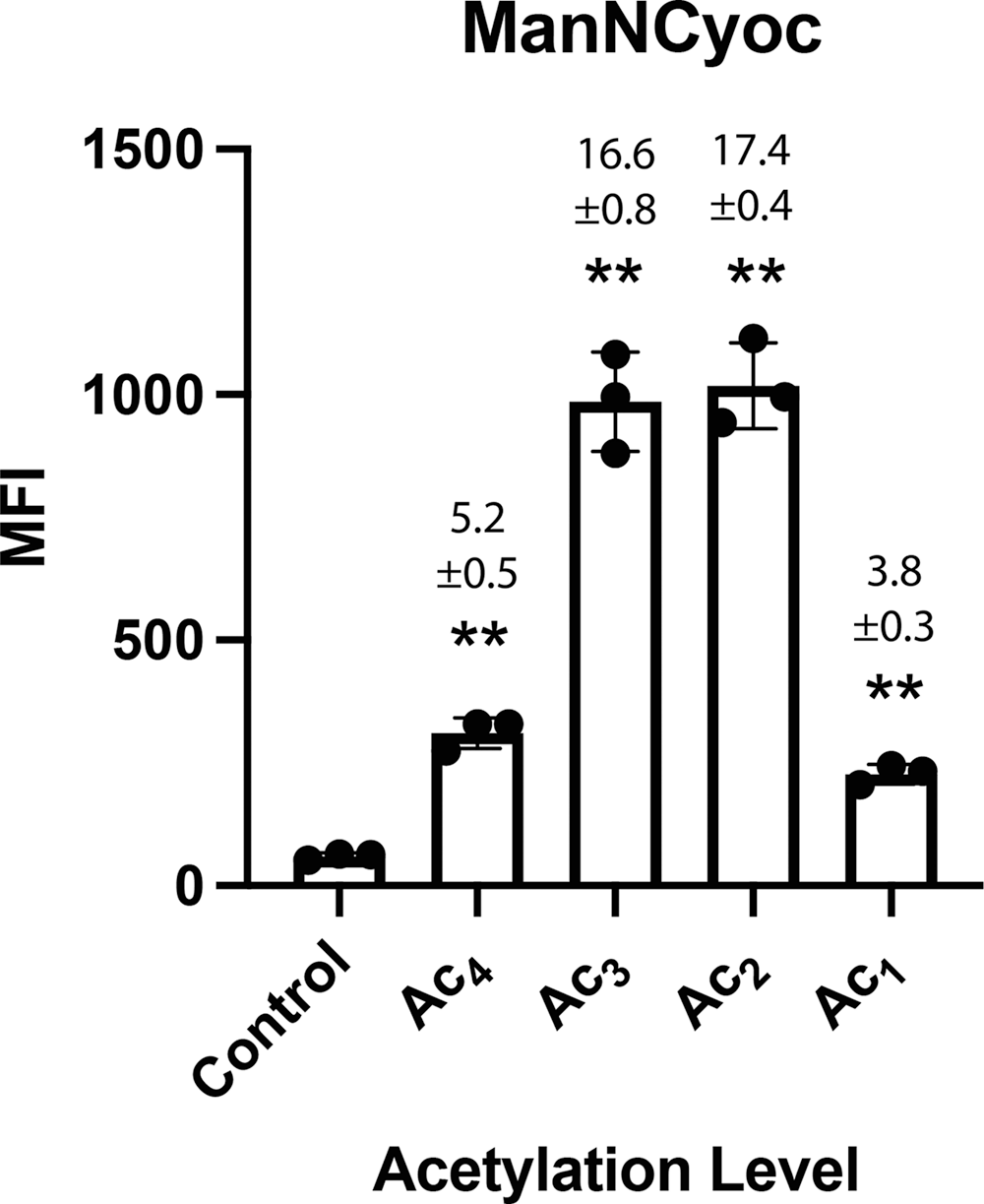
Glycan labeling with Ac*_x_*ManNCyoc. COLO205 cells were incubated in the presence or absence (control) of the indicated Ac*_x_*ManNCyoc sugar at 125 μM (Ac_1_, Ac_2_, Ac_3_, or Ac_4_) for 24 h. They were then incubated with dyes Tz-PEG_11_-AlexaFluor647 (5 μM) + Sytox green cell death stain (50 nM) for 1 h at 37 °C and analyzed by flow cytometry. Median fluorescence intensity (MFI) is shown as mean ± SD. Signal-to-background ratios (SBRs) relative to the control (PBS) are shown above each bar; *n* = 3 technical replicates. Statistical analysis was performed using an unpaired *t* test with Welch correction (****P ≤ 0.0001, ***P ≤ 0.001, **P ≤ 0.01, *P ≤ 0.05).

The insolubility of the tetraacetylated cyclopropene sugars was immediately apparent, with visible turbidity occurring upon addition of a solution Ac_4_ManNCyoc in DMSO to the culture medium. For the less acetylated sugars, this was not observed. Figure 6 shows a flow cytometric analysis of metabolic labeling with Ac*_x_*ManNCyoc, as a function of the degree of acetylation of the sugar (Ac_1_–Ac_4_) in COLO205 cells. The median fluorescence intensity (MFI) was (17.4 ± 0.4)- and (16.6 ± 0.8)-fold higher than the control (PBS) for the di- and triacetylated compounds, respectively. In contrast, Ac_4_ManNCyoc-treated cells showed a much-reduced signal intensity, which can be explained by the poor aqueous solubility of this peracetylated monosaccharide. Ac_1_ManNCyoc also showed decreased signal intensity, presumably due to its reduced cell-membrane permeability. Ac_3_ManNCyoc and Ac_2_ManNCyoc are therefore better candidates for imaging of tumor hypersialylation than the widely used tetraacetylated derivative. These results contrast with earlier work,^41^ which showed that for ManNAc (without any bio-orthogonal tag) increased sialic acid production was observed with more hydrophobic hydroxyl protecting groups than acetyl.

**Figure 7:**
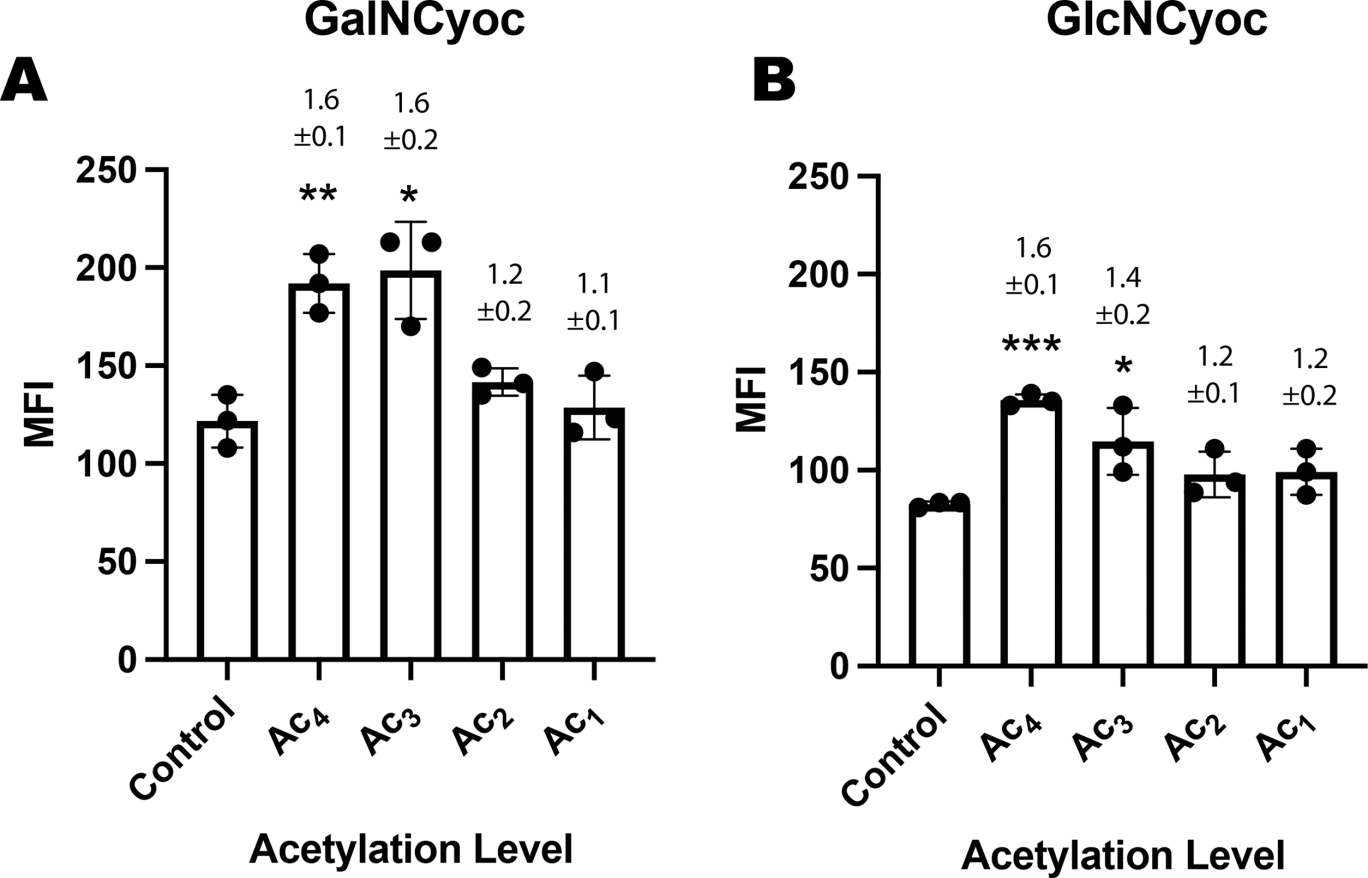
Glycan labeling with Ac*_x_*GalNCyoc and Ac*_x_*GlcNCyoc. COLO205 cells were incubated in the presence or absence of the indicated sugar (A) GalNCyoc and (B) GlcNCyoc 125 μM (Ac_1_, Ac_2_, Ac_3_, or Ac_4_) for 24 h. They were then incubated with dyes Tz-PEG_11_-AlexaFluor647 (5 μM) + Sytox green cell death stain (50 nM) for 1 h at 37 °C and analyzed by flow cytometry. Median fluorescence intensity (MFI) is shown as mean ± SD. Signal-to-background ratios (SBRs) relative to the control (PBS) are above each bar; *n* = 3 technical replicates. Statistical analysis was performed using an unpaired *t* test with Welch correction (****P ≤ 0.0001,***P ≤ 0.001, **P ≤ 0.01, *P ≤ 0.05).

The same methodology was used with the Ac*_x_*GalNCyoc and Ac*_x_*GlcNCyoc derivatives (Figure 7). However, the same effect was not observed for these monosaccharides. Instead, tetra- and triacetylated derivatives produced moderate SBRs of (1.6 ± 0.1) and (1.6 ± 0.2) for Ac_4_GalNCyoc and Ac_3_GalNCyoc, respectively, and (1.6 ± 0.1) and (1.4 ± 0.2) for Ac_4_GlcNCyoc and Ac_3_GlcNCyoc, respectively, whereas lower acetylation levels resulted in no significant sugar incorporation. Therefore, in this case, it is not solubility that limits the incorporation of the monosaccharide into the cell surface glycans but the inherently low labeling efficiency with these sugars. The significantly lower labeling with Ac_4_GalNCyoc and Ac_4_GlcNCyoc than with Ac_4_ManNCyoc has been observed previously in human embryonic kidney cells (HEK293T).^23,42^ Poor labeling with other tagged GalN and GlcN analogues (relative to their ManN counterparts) has also been reported previously.^43,44^ Monosaccharides can be interconverted intracellularly to some extent by epimerases and so it is possible that some Ac*_x_*GlcNCyoc is eventually expressed on the cell surface as sialic acid residues.^45^

**Figure 8:**
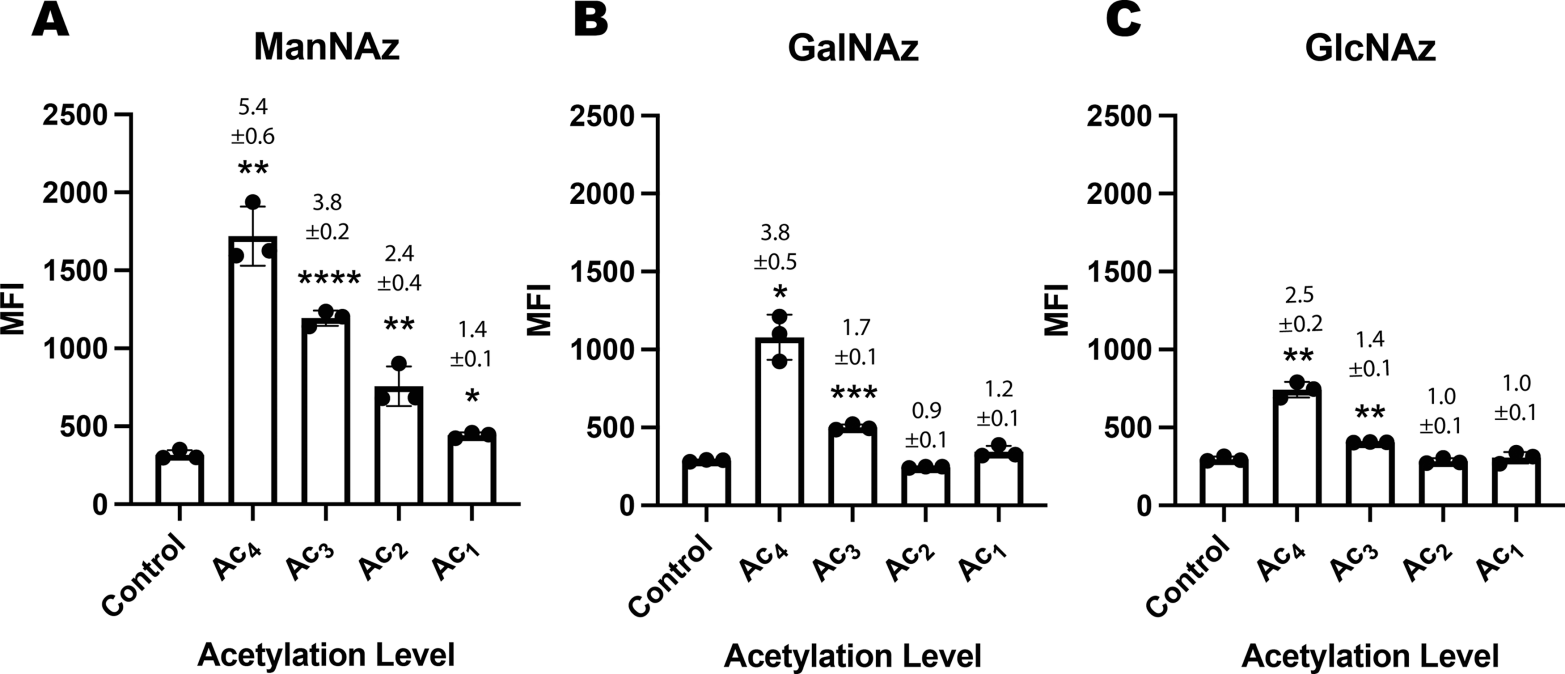
Glycan labeling with azide-tagged sugars. COLO205 cells were incubated in the presence or absence of the appropriate sugar, (A) ManNAz, (B) GalNAz, and (C) GlcNAz 125 μM (Ac_1_, Ac_2_, Ac_3_, or Ac_4_), for 24 h. They were then incubated with dyes TMDIBO-Lys-AlexaFluor647 (30 μM) + Sytox green cell death stain (50 nM) for 1 h at 37 °C and analyzed by flow cytometry. Median fluorescence intensity (MFI) is shown as mean ± SD. Signal-to-background ratios (SBRs) relative to the control (PBS) are above each bar; *n* = 3 technical replicates. Statistical analysis was performed using an unpaired *t* test with Welch correction (****P ≤ 0.0001, ***P ≤ 0.001, **P ≤ 0.01, *P ≤ 0.05).

Tetra-acetylated azido-tagged sugars have been widely used for MOE both *in vitro* and *in vivo*,^28−30^ but it has not been shown that tetra-acetylation is the optimum level of acetylation. When the variably acetylated azido-tagged sugars were tested on COLO205 cells, the tetra-acetylated derivatives showed the best labeling efficiency (Figure 8). Reduced incorporation of Ac*_x_*GalNAz and Ac*_x_*GlcNAz relative to Ac*_x_*ManNAz was again observed as previously demonstrated for the cyclopropene-tagged sugars (Figures 6 and 7).

We believe that the difference between the ManNCyoc derivatives (where the di- and triacetylated sugars were better incorporated) and the ManNAz derivatives (where the tetra-acetylated sugar is the best incorporated) is due to the greater inherent solubility of Ac_4_ManNAz. This is supported by its much lower *c* log *P* value relative to Ac_4_ManNCyoc (Figure 2). Therefore, the solubility of Ac_4_ManNAz does not limit its incorporation and lower levels of acetylation reduce the labeling, presumably due to reduced cell permeability. There is, in fact, evidence in the literature that the permeability of Ac_4_ManNAz is less than ideal as the tetrabutanoylated Bu_4_ManNAz is better incorporated.^46^ However, Bu_4_ManNAz may be too nonpolar as 1,3,4-Bu_3_ManNAz is incorporated better still.^7,46^

Recent reports^47,48^ of nonenzymic *S*-glyco modification of proteins by anomerically deacetylated monosaccharides are not thought to explain the increase in labeling with Ac_3_ManNCyoc and Ac_2_ManNCyoc (relative to Ac_4_ManNCyoc). If the labeling increase was due to *S*-glyco modification, the same effect would have been expected in other di- and triacetylated sugars (e.g., GalNCyoc and GlcNCyoc) since GalN and GlcN derivatives can also be substrates for *S*-glyco modification. All three azido-tagged sugars have been shown to participate in this modification,^47,48^ and in this study, none of the lower degrees of acetylation of the azido sugars showed enhanced labeling. Since only Ac_3_ManNCyoc and Ac_2_ManNCyoc show increased labeling relative to the tetraacetylated derivative, this nonenzymatic reaction is unlikely to be responsible for the enhanced labeling.

**Figure 9:**
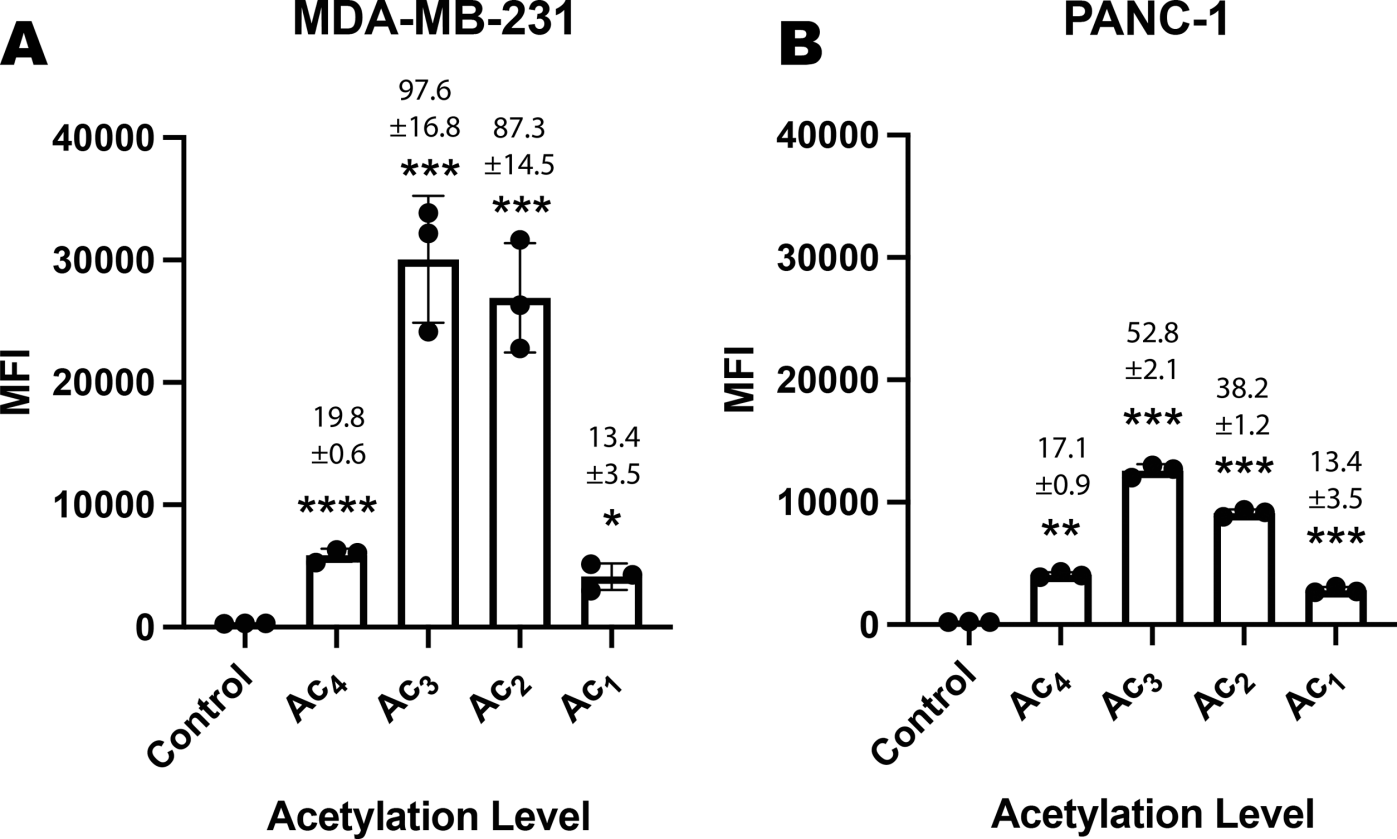
Glycan labeling of other cell lines with Ac*_x_*ManNCyoc. (A) MDA-MB-321 and (B) PANC-1 cells were incubated in the presence or absence (control) of the appropriate Ac*_x_*ManNCyoc sugar 125 μM (Ac_1_, Ac_2_, Ac_3_, or Ac_4_) for 24 h. They were then incubated with dyes Tz-PEG_11_-AlexaFluor647 (5 μM) + Sytox green cell death stain (50 nM) for 1 h at 37 °C and analyzed by flow cytometry. Median fluorescence intensity (MFI) is shown as mean ± SD. Signal-to-background ratios (SBRs) relative to the control (PBS) are above each bar; *n* = 3 technical replicates. Statistical analysis was performed using an unpaired *t* test with Welch correction (****P ≤ 0.0001, ***P ≤ 0.001, **P ≤ 0.01, *P ≤ 0.05).

Next, we determined whether the enhanced cell labeling we had observed with partially acetylated Ac*_x_*ManNCyoc in COLO205 cells was replicated in other tumor cell lines. MDA-MB-231, an invasive triple-negative epithelial human breast cancer cell line, and PANC-1, a pancreatic carcinoma cell line, were selected. For both cell lines, the extent of labeling was significantly increased at all levels of acetylation compared to COLO205 cells (Figure 9). The increase in labeling with the tri- and diacetylated ManNCyoc derivatives compared to the tetra-acetylated derivative was further evidence that solubility can become a limiting factor when the tagged sugars show high levels of incorporation

**Figure 10:**
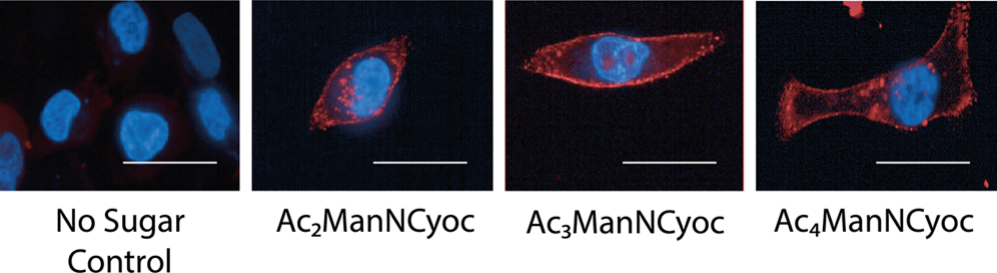
Epifluorescence microscopy of labeled MDA-MB-231 cell glycans. Cells were cultured with or without Ac*_x_*ManNCyoc (125 μM) for 24 h and then incubated with Tz-PEG_11_-AlexaFluor647 (5 μM) and DAPI (100 nM) nuclear stain for 15 min at 37 °C. Red: AlexaFluor647, blue: DAPI. Scale bar: 50 μm.

Epifluorescence microscopy confirmed these findings. MDA-MB-231 cells were incubated with either Ac_2_ManNCyoc, Ac_3_ManNCyoc, Ac_4_ManNCyoc (125 μM for 24 h at 37 °C), or vehicle (PBS). Visualization of the cells after incubation with Tz-PEG_11_-AlexaFluor647 (and DAPI) showed strong labeling of the sugar-treated cells (Figure 10). Vehicle-treated cells showed very little nonspecific labeling, confirming that Ac_3_ManNCyoc is a promising tool for imaging tumor hypersialylation. No significant effect on cell viability with Ac_3_ManNCyoc addition was seen at 125 μM (Figure S1.3).

## Conclusions

Ac_3_ManNCyoc and Ac_2_ManNCyoc are novel candidates for probing tumor hypersialylation using metabolic labeling and subsequent imaging. They represent the optimal compromise between sugar solubility and membrane permeability. Ac_3_ManNCyoc is the preferred choice, as it is the more easily synthesized of the two compounds and is a single compound.

It is notable that Ac_3_ManNCyoc yielded a threefold greater signal-to-background ratio than Ac_4_ManNAz and could, therefore, be considered the monosaccharide of choice for sialic acid imaging. We believe that this improvement in labeling is probably due to the faster IED-DA reaction of the Cyoc group^23^ (ca. 1 M^–1^ s^–1^) compared with the SPAAC reaction of the azide^49^ (ca. 0.1 M^–1^ s^–1^), meaning that a higher percentage of the cell-surface tags get ligated to the fluorophore. The principle, demonstrated here, that larger tags can, in some cases, lead to better labeling, if they have faster rates of bio-orthogonal reaction and if the right balance of water-solubility versus hydrophobicity is achieved, may apply much more generally in metabolic labeling of all kinds of biomolecules.

## Supporting Information Available

Procedures for the synthesis of chemicals and analytical data; procedures for cell-labeling studies and results from flow-cytometry, microscopy, and cell-viability studies (PDF)

## References

[ref1] Hart G. W.; Copeland R. J. Glycomics hits the big time. Cell 2010, 143, 672–676. 10.1016/j.cell.2010.11.008. 21111227 PMC3008369

[ref2] Freeze H. H. Genetic defects in the human glycome. Nat. Rev. Genet. 2006, 7, 537–551. 10.1038/nrg1894. 16755287

[ref3] Fuster M. M.; Esko J. D. The sweet and sour of cancer: glycans as novel therapeutic targets. Nat. Rev. Cancer 2005, 5, 526–542. 10.1038/nrc1649. 16069816

[ref4] Stowell S. R.; Ju T.; Cummings R. D. Protein glycosylation in cancer. Annu. Rev. Pathol.: Mech. Dis. 2015, 10, 473–510. 10.1146/annurev-pathol-012414-040438. PMC4396820 25621663

[ref5] Dube D. H.; Bertozzi C. R. Glycans in cancer and inflammation — potential for therapeutics and diagnostics. Nat. Rev. Drug Discovery 2005, 4, 477–488. 10.1038/nrd1751. 15931257

[ref6] Pinho S. S.; Reis C. A. Glycosylation in cancer: mechanisms and clinical implications. Nat. Rev. Cancer 2015, 15, 540–555. 10.1038/nrc3982. 26289314

[ref7] Agatemor C.; Buettner M. J.; Ariss R.; Muthiah K.; Saeui C. T.; Yarema K. J. Exploiting metabolic glycoengineering to advance healthcare. Nat. Rev. Chem. 2019, 3, 605–620. 10.1038/s41570-019-0126-y. 31777760 PMC6880190

[ref8] Taniguchi N.; Kizuka Y. Glycans and cancer: role of N-glycans in cancer biomarker, progression and metastasis, and therapeutics. Adv. Cancer Res. 2015, 126, 11–51. 10.1016/bs.acr.2014.11.001. 25727145

[ref9] Kufe D. W. Mucins in cancer: function, prognosis and therapy. Nat. Rev. Cancer 2009, 9, 874–885. 10.1038/nrc2761. 19935676 PMC2951677

[ref10] Bhide G. P.; Colley K. J. Sialylation of N-glycans: mechanism, cellular compartmentalization and function. Histochem. Cell Biol. 2017, 147, 149–174. 10.1007/s00418-016-1520-x. 27975143 PMC7088086

[ref11] Moons S. J.; Adema G. J.; Derks M. T. G. M.; Boltje T. J.; Büll C. Sialic acid glycoengineering using N-acetylmannosamine and sialic acid analogs. Glycobiology 2019, 29, 433–445. 10.1093/glycob/cwz026. 30913290

[ref12] Büll C.; Stoel M. A.; Den Brok M. H.; Adema G. J. Sialic acids sweeten a tumor’s life. Cancer Res. 2014, 74, 3199–3204. 10.1158/0008-5472.CAN-14-0728. 24830719

[ref13] Dube D. H.; Bertozzi C. R. Metabolic oligosaccharide engineering as a tool for glycobiology. Curr. Opin. Chem. Biol. 2003, 7, 616–625. 10.1016/j.cbpa.2003.08.006. 14580567

[ref14] Saxon E.; Bertozzi C. R. Cell surface engineering by a modified Staudinger reaction. Science 2000, 287, 2007–2010. 10.1126/science.287.5460.2007. 10720325

[ref15] Oliveira B. L.; Guo Z.; Bernardes G. J. L. Inverse electron demand Diels-Alder reactions in chemical biology. Chem. Soc. Rev. 2017, 46, 4895–4950. 10.1039/C7CS00184C. 28660957

[ref16] Jewett J. C.; Bertozzi C. R. Cu-free click cycloaddition reactions in chemical biology. Chem. Soc. Rev. 2010, 39, 1272–1279. 10.1039/b901970g. 20349533 PMC2865253

[ref17] Stairs S.; Neves A. A.; Stöckmann H.; Wainman Y. A.; Ireland-Zecchini H.; Brindle K. M.; Leeper F. J. Metabolic glycan imaging by isonitrile-tetrazine click chemistry. ChemBioChem. 2013, 14, 1063–1067. 10.1002/cbic.201300130. 23670994 PMC3743162

[ref18] Späte A.-K.; Bußkamp H.; Niederwieser A.; Schart V. F.; Marx A.; Wittmann V. Rapid labeling of metabolically engineered cell-surface glycoconjugates with a carbamate-linked cyclopropene reporter. Bioconjugate Chem. 2014, 25, 147–154. 10.1021/bc4004487. 24328258

[ref19] Schart V. F.; Hassenrück J.; Späte A.-K.; Dold J. E. G. A.; Fahrner R.; Wittmann V. Triple orthogonal labeling of glycans by applying photoclick chemistry. ChemBioChem. 2019, 20, 166–171. 10.1002/cbic.201800740. 30499611

[ref20] Hudak J. E.; Alvarez D.; Skelly A.; Andrian U. H. v.; Kasper D. L. Illuminating vital surface molecules of symbionts in health and disease. Nat. Microbiol. 2017, 2, 17099 10.1038/nmicrobiol.2017.99. 28650431 PMC5546223

[ref21] Patterson D. M.; Nazarova L. A.; Xie B.; Kamber D. N.; Prescher J. A. Functionalized cyclopropenes as bioorthogonal chemical reporters. J. Am. Chem. Soc. 2012, 134, 18638–18643. 10.1021/ja3060436. 23072583

[ref22] Xiong D.-C.; Zhu J.; Han M.-J.; Luo H.-X.; Wang C.; Yu Y.; Ye Y.; Tai G.; Ye X.-S. Rapid probing of sialylated glycoproteins in vitro and in vivo via metabolic oligosaccharide engineering of a minimal cyclopropene reporter. Org. Biomol. Chem. 2015, 13, 3911–3917. 10.1039/C5OB00069F. 25735895

[ref23] Hassenrück J.; Wittmann V. Cyclopropene derivatives of aminosugars for metabolic glycoengineering. Beilstein J. Org. Chem. 2019, 15, 584–601. 10.3762/bjoc.15.54. 30931000 PMC6423581

[ref24] Yang J.; Šečkute J.; Cole C. M.; Devaraj N. K. Live-cell imaging of cyclopropene tags with fluorogenic tetrazine cycloadditions. Angew. Chem., Int. Ed. 2012, 51, 7476–7479. 10.1002/anie.201202122. PMC3431913 22696426

[ref25] Cole C. M.; Yang J.; Šečkutė J.; Devaraj N. K. Fluorescent Live-Cell Imaging of Metabolically Incorporated Unnatural Cyclopropene-Mannosamine Derivatives. ChemBioChem 2013, 14, 205–208. 10.1002/cbic.201200719. 23292753 PMC4010072

[ref26] Patterson D. M.; Jones K. A.; Prescher J. A. Improved cyclopropene reporters for probing protein glycosylation. Mol. BioSyst. 2014, 10, 1693–1697. 10.1039/c4mb00092g. 24623192

[ref27] Luchansky S. J.; Hang H. C.; Saxon E.; Grunwell J. R.; Yu C.; Dube D. H.; Bertozzi C. R. Constructing azide-labeled cell surfaces using polysaccharide biosynthetic pathways. Methods Enzymol. 2003, 362, 249–272. 10.1016/s0076-6879(03)01018-8. 12968369

[ref28] Prescher J. A.; Dube D. H.; Bertozzi C. R. Chemical remodelling of cell surfaces in living animals. Nature 2004, 430, 873–877. 10.1038/nature02791. 15318217

[ref29] Baskin J. M.; Prescher J. A.; Laughlin S. T.; Agard N. J.; Chang P. V.; Miller I. A.; Lo A.; Codelli J. A.; Bertozzi C. R. Copper-free click chemistry for dynamic in vivo imaging. Proc. Natl. Acad. Sci. U.S.A. 2007, 104, 16793–16793. 10.1073/pnas.0707090104. 17942682 PMC2040404

[ref30] Neves A. A.; Wainman Y. A.; Wright A.; Kettunen M. I.; Rodrigues T. B.; McGuire S.; Hu D.-E.; Bulat F.; Geninatti Crich S.; Stöckmann H.; Leeper F. J.; Brindle K. M. Imaging glycosylation in vivo by metabolic labeling and magnetic resonance imaging. Angew. Chem., Int. Ed. 2016, 55, 1286–1290. 10.1002/anie.201509858. PMC4737346 26633082

[ref31] Molinspiration Cheminformatics, 2021. https://www.molinspiration.com/cgi-bin/properties.

[ref32] Laughlin S. T.; Bertozzi C. R. Metabolic labeling of glycans with azido sugars and subsequent glycan-profiling and visualization via Staudinger ligation. Nat. Protoc. 2007, 2, 2930–2944. 10.1038/nprot.2007.422. 18007630

[ref33] Ren B.; Zhang L.; Zhang M. Progress on selective acylation of carbohydrate hydroxyl groups. Asian J. Org. Chem. 2019, 8, 1813–1823. 10.1002/ajoc.201900400.

[ref34] Khan R.; Konowicz P. A.; Gardossi L.; Matulová M.; De Gennaro S. Regioselective deacetylation of fully acetylated mono- and di-saccharides with hydrazine hydrate. Aust. J. Chem. 1996, 49, 293–298. 10.1071/CH9960293.

[ref35] Mikamo M. Facile 1-O-deacylation of per-O-acylaldoses. Carbohydr. Res. 1989, 191, 150–153. 10.1016/0008-6215(89)85056-6.

[ref36] Kaya E.; Sonmez F.; Kucukislamoglu M.; Nebioglu M. Selective anomeric deacetylation using zinc acetate as catalyst. Chem. Pap. 2012, 66, 312–315. 10.2478/s11696-012-0143-5.

[ref37] Fiandor J.; Garciá-López M. T.; De Las Heras F. G.; Méndez-Castrillón P. P. A Facile Regioselective 1-O-deacylation of peracylated glycopyranoses. Synthesis 1985, 1985, 1121–1123. 10.1055/s-1985-31446.

[ref38] Stöckmann H.; Neves A. A.; Day H. A.; Stairs S.; Brindle K. M.; Leeper F. J. (E,E)-1,5-Cyclooctadiene: a small and fast click-chemistry multitalent. Chem. Commun. 2011, 47, 7203–7205. 10.1039/c1cc12161h. 21611648

[ref39] Neves A. A.; Stöckmann H.; Wainman Y. A.; Kuo J. C. H.; Fawcett S.; Leeper F. J.; Brindle K. M. Imaging cell surface glycosylation in vivo using ″double click″ chemistry. Bioconjugate Chem. 2013, 24, 934–941. 10.1021/bc300621n. PMC3687584 23642228

[ref40] Neves A. A.; Stöckmann H.; Harmston R. R.; Pryor H. J.; Alam I. S.; Ireland-Zecchini H.; Lewis D. Y.; Lyons S. K.; Leeper F. J.; Brindle K. M. Imaging sialylated tumor cell glycans in vivo. FASEB J. 2011, 25, 2528–2537. 10.1096/fj.10-178590. 21493886

[ref41] Kim E. J.; Sampathkumar S.-G.; Jones M. B.; Rhee J. K.; Baskaran G.; Goon S.; Yarema K. J. Characterization of the Metabolic Flux and Apoptotic Effects of O-Hydroxyl- and N-Acyl-modified N-Acetylmannosamine Analogs in Jurkat Cells. J. Biol. Chem. 2004, 279, 18342–18352. 10.1074/jbc.M400205200. 14966124

[ref42] Späte A.-K.; Schart V. F.; Häfner J.; Niederwieser A.; Mayer T. U.; Wittmann V. Expanding the scope of cyclopropene reporters for the detection of metabolically engineered glycoproteins by Diels-Alder reactions. Beilstein J. Org. Chem. 2014, 10, 2235–2242. 10.3762/bjoc.10.232. 25298790 PMC4187077

[ref43] Vocadlo D. J.; Hang H. C.; Kim E. J.; Hanover J. A.; Bertozzi C. R. A chemical approach for identifying O-GlcNAc-modified proteins in cells. Proc. Natl. Acad. Sci. U.S.A. 2003, 100, 9116–9121. 10.1073/pnas.1632821100. 12874386 PMC171382

[ref44] Campbell C. T.; Sampathkumar S. G.; Yarema K. J. Metabolic oligosaccharide engineering: perspectives, applications, and future directions. Mol. BioSyst. 2007, 3, 187–194. 10.1039/b614939c. 17308665

[ref45] Dold J. E. G. A.; Wittmann V. Metabolic glycoengineering with azide-and alkene-modified hexosamines: quantification of sialic acid levels. ChemBioChem 2021, 22, 1243–1251. 10.1002/cbic.202000715. 33180370 PMC8048827

[ref46] Almaraz R. T.; Aich U.; Khanna H. S.; Tan E.; Bhattacharya R.; Shah S.; Yarema K. J. Metabolic Oligosaccharide Engineering With N-Acyl Functionalized ManNAc Analogs: Cytotoxicity, Metabolic Flux, and Glycan-Display Considerations. Biotechnol. Bioeng. 2012, 109, 992–1006. 10.1002/bit.24363. 22068462 PMC3288793

[ref47] Qin W.; Qin K.; Fan X.; Peng L.; Hong W.; Zhu Y.; Lv P.; Du Y.; Huang R.; Han M.; Cheng B.; Liu Y.; Zhou W.; Wang C.; Chen X. Artificial cysteine S-glycosylation induced by per-O-acetylated unnatural monosaccharides during metabolic glycan labeling. Angew. Chem., Int. Ed. 2018, 57, 1817–1820. 10.1002/anie.201711710. 29237092

[ref48] Qin K.; Zhang H.; Zhao Z.; Chen X. Protein S-glyco-modification through an elimination-addition mechanism. J. Am. Chem. Soc. 2020, 142, 9382–9388. 10.1021/jacs.0c02110. 32339456

[ref49] Stöckmann H.; Neves A. A.; Stairs S.; Ireland-Zecchini H.; Brindle K. M.; Leeper F. J. Development and evaluation of new cyclooctynes for cell surface glycan imaging in cancer cells. Chem. Sci. 2011, 2, 932–936. 10.1039/c0sc00631a. 22724056 PMC3378185

