# Supplementary material for: Metabolic Glycan Labeling of Cancer Cells Using Variably Acetylated Monosaccharides
Source: Bioconjug Chem. 2022 Jul 25;33(8):1467–73. doi: 10.1021/acs.bioconjchem.2c00169 (PMC9389531; doi:10.1021/acs.bioconjchem.2c00169)

Supporting Information

Metabolic Glycan Labelling of Cancer Cells using Variably Acetylated Monosaccharides

Daniel R. Parle,[a] Flaviu Bulat,[b] Shahd Fouad,[a] Heather Zecchini,[b] Kevin M. Brindle,[b] André A. Neves,[b] and Finian J. Leeper[a]*

a. *Yusuf Hamied Department of Chemistry, University of Cambridge, Lensfield Road, Cambridge CB2 1EW, United Kingdom*
b. *Cancer Research UK Cambridge Institute, University of Cambridge, Li Ka Shing Centre, Robinson Way, Cambridge CB2 0RE, United Kingdom*

Contents

1. **Experimental Section**
   - **1.1 General Information** S-2
   - **1.2 Chemical Synthesis** S-2
   - **1.3 *In Vitro* Experiments**
     - **Flow Cytometry method and histograms** S-5
     - **Fluorescent microscopy method and images** S-7
     - **Cell viability and growth methods and data** S-8
   - **1.4 References** S-8

2. **^{1}H and ^{13}C NMR Spectra**
   - **2.1 1D Spectra** S-9
   - **2.2 2D Spectra** S-21

1. Experimental Section

1.1 General Information

All commercially obtained chemicals were used without further purification. NMR spectra were recorded on a 400 MHz Avance III HD Smart Probe Spectrometer. 1H NMR spectra were obtained at 400 MHz and 13C NMR at 100 MHz. Chemical shift values are given in ppm and coupling constants in Hz. Multiplicity is indicated as follows s (singlet); d (doublet); t (triplet); q (quartet); m (multiplet), dd (doublet of doublets); etc. Solvent was removed on a Büchi rotary evaporator. TLC was conducted on Merck Kieselgel 60 F254. TLC plates were visualised with ceric ammonium molybdate and permanganate stains. Flash chromatography was carried out using a CombiFlash Rf+ system with RediSep Rf columns (0.040-0.063 mm). HRMS were recorded on a Waters Acquity UPLC system running in positive mode with an electrospray source.

The culture conditions for the various cell lines are as follows. All cells were incubated at 37 °C under 5% CO_2. The culture medium used for COLO205 cells was RPMI 1640 21875-034, and for MDA-MB-231/PANC-1 cells Dulbecco's Modified Eagle's Medium (DMEM), 41966-029. All media were supplemented with 10% foetal bovine serum and supplied by Gibco™Therrmo Fisher. A Vi-CELL™ Cell Viability Analyzer was used to assess cell viability and determine cell seeding density.

1.2 Chemical Synthesis

ManNAz, GalNAz and GlcNAz fully acetylated and fully deacetylated were synthesised as previously described.[1]

ManNCyoc, GalNCyoc and GlcNCyoc fully acetylated and fully deacetylated were synthesised as previously described.[2]

General Procedure for Mono/di-acetylated sugars

The unacetylated tagged sugars (ManNAz, GalNAz, GlcNAz, ManNCyoc, GalNCyoc and GlcNCyoc) (1 eq.) were dissolved in pyridine (0.1 M). Acetic anhydride (1 or 2 eq.) dissolved in pyridine (1 mL) was added dropwise. The reaction mixture was stirred at room temperature overnight, then concentrated *in vacuo*. The resulting residue was purified using flash chromatography eluting with 5-10% MeOH in DCM.

Mono and di-acetylated derivatives were obtained as a mixture of different degrees of acetylation. **Section 2** contains spectra showing how the major degree of acetylation was assigned using ^{1}H NMR. The major level of acetylation for cyclopropene tagged sugars was determined by integration of the ^{1}H peaks for the acetyl groups (~2.1 ppm) relative to the alkene proton of the cyclopropene (~6.7 ppm) – note that the peak at ca. 2.17 ppm is the methyl group on the cyclopropene. For the azido sugars, the major level was assigned by comparison of the integral of the main anomeric signal with the acetyl peaks. The level of acetylation was also confirmed using HRMS.

Azido Tagged sugars

HRMS: (ESI+) **AcManNAz**: 327.0814, **AcGalNAz:** 327.0850, **AcGlcNAz:** 327.0904 ($C_{10}H_{16}N_4O_7.Na^+$, requires 327.0911).
Ac_2ManNAz: 369.1017, **Ac_2GalNAz:** 369.1044, **Ac_2GlcNAz:** 369.1026 ($C_{12}H_{18}N_4O_8.Na^+$, requires 369.1017).

Cyclopropene Tagged sugars

HRMS: (ESI+) **AcManNCyoc**: 354.1162, **AcGalNCyoc:** 354.1164, **AcGlcNCyoc:** 354.1165. ($C_{14}H_{21}NO_8.Na^+$, requires 354.1159).
Ac_2ManNCyoc: 396.1266, **Ac_2GalNCyoc:** 396.1270, **Ac_2GlcNCyoc:** 396.1267 ($C_{12}H_{18}N_4O_8.Na^+$, requires 396.1265).

General Procedure for Triacetylated Sugars

The fully acetylated tagged sugars (Ac_4ManNAz, Ac_4GalNAz, Ac_4GlcNAz, Ac_4ManNCyoc, Ac_4GalNCyoc and Ac_4GlcNCyoc) were dissolved in THF (0.1 M) and 7M ammonia in MeOH (10 eq.) added. The reaction mixture was stirred at room temperature for 2 h and then concentrated *in vacuo*. The resulting brown residue was purified by flash chromatography eluting with 50% EtOAc in hexanes.

Azido Tagged Sugars

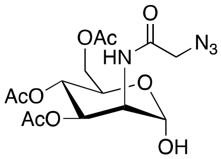

Ac_3ManNAz: (42% yield) δ_H (400 MHz, CD_3OD) 5.33 (dd, *J* = 10.0, 4.5 Hz, 1H), 5.18 (d, *J* = 10.0 Hz, 1H), 5.07-5.11 (m, 1H), 4.54 (dd, *J* = 4.5, 1.5 Hz, 1H), 4.24-4.32 (m, 2H), 4.08-4.14 (m, 2H), 3.97 (d, J = 4.0 Hz, 1H), 2.08 (s, 3H), 2.07 (s, 3H), 1.98 (s, 3H).
δ_C (100 MHz, CD_3OD) 172.6, 171.89, 171.85, 170.8, 94.6, 71.1, 69.2, 68.0, 64.4, 52.65, 52.64, 23.8, 20.9, 20.8.

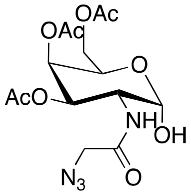

Ac_3GalNAz: (50% yield) δ_H (400 MHz, CD_3OD) 5.42-5.44 (m, 1H), 5.26 (dd, *J* = 11.3, 3.3 Hz), 5.22 (d, *J* = 3.3 Hz), 4.41-4.51 (m, 2H), 4.10-4.21 (m, 2H), 2.17 (s, 3H), 2.04 (s, 3H), 1.97 (s, 3H).
δ_C (100 MHz, CD_3OD) 170.8, 170.8, 170.6, 169.0, 91.5, 70.5, 68.4, 67.7, 65.8, 61.8, 51.3, 19.3, 19.2, 19.2.

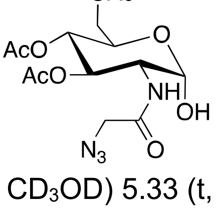

Ac_3GlcNAz: (43% yield) δ_H (400 MHz, CD_3OD) 5.33 (t, *J* = 10.2, 1H, 3-H), 5.15 (d, *J* = 3.4 Hz, 1H, 1-H), 5.04 (t, *J* = 10.2, 1H, 4-H), 4.24-4.30 (m, 2H, 5-H & 6-H_A), 4.22 (dd, *J* = 10.5 & 3.4, 1H, H-2) 4.10 (d, *J* = 11.0, 1H, 6-H_B), 3.87 (s, 2H, CH_2N_3), 2.07 (s, 3H, 6-OAc), 2.03 (s, 3H, 4-OAc) & 2.00 (s, 3H, 3-OAc).
δ_C (100 MHz, CD_3OD) 172.6 (6-OCO), 172.4 (3-OCO), 171.5 (4-OCO), 170.4 (2-NHCO), 92.6 (C-1), 72.6 (C-3), 70.5 (C-4), 68.5 (C-5), 63.7 (C-6), 53.8 (C-2), 52.8 (CH_2N_3), 20.82 (4-OAc), 20.77 (3-OAc), 20.76 (6-OAc).

^{1}H and ^{13}C assignments were made using COSY, HSQC and HMBC spectra.

HRMS: (ESI+) Ac$_3$ManNAz: 411.1120, Ac$_3$GalNAz: 411.1125, Ac$_3$GlcNAz: 411.1140. ($C_{12}H_{18}N_4O_8.Na^+$, requires 411.1122).

<u>Cyclopropene Tagged sugars</u>

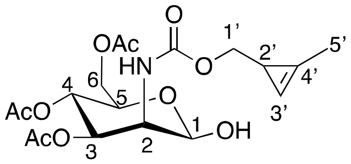

Ac$_3$ManNCyoc: The following assignments were confirmed using COSY, HSQC and HMBC spectra. Note, because the (2-methylcycloprop-2-en-1-yl)methanol used to synthesise this compound was racemic, there are two diastereoisomers differing in the stereochemistry of the chiral centre of the cyclopropene ring. The existence of two isomers was not evident for any of the mannosamine peaks but was seen for some of the (2-methylcycloprop-2-en-1-yl)methyl peaks. Where this is the case, the different isomers have been distinguished with prime (') and double-prime (").

Ac$_3$ManNCyoc: (27% yield) δ_H (400 MHz, CD_3OD) 6.67 (br s, 1H, 3'-H), 5.31 (dd, *J* = 10.2, 4.4 Hz, 1H, 3-H), 5.21 (t, *J* = 10.2 Hz, 1H, 4-H), 5.08 (br s, 1H, 1-H), 4.29 (dd, *J* = 11.8, 6.0 Hz, 1H, 6-H_A), 4.19-4.23 (m, 2H, 2 & 5-H), 4.10 (dd, *J* = 11.8, 2.5 Hz, 1H, 6-H_B), 4.01 (dd, *J* = 11.4, 4.7 Hz, 0.5H, 1'-H_A), 3.96 (dd, *J* = 11.0, 5 Hz, 0.5H, 1"-H_A), 3.89 (dd, *J* = 11.3 & 5.2 Hz, 0.5H, 1"-H_B), 3.83 (dd, *J* = 11.0 & 5.6 Hz, 0.5H, 1'-H_B), 2.16 (s, 3H, 5'-H), 2.08, 2.07 and 1.98 (each 3H, s, OAc), 1.67 (br t, *J* = 5.0 Hz, 1H, 2'-H).
δ_C (100 MHz, CD_3OD) 172.6, 171.9 & 171.8 (Me*C*OO), 159.4 (NHCOO), 122.1 (C-4'), 103.1 & 103.0 (C-3' & 3"), 95.1 (C-1), 73.9 (C-1') & 73.6 (C-1"), 71.5 (C-3), 69.3 (C-5), 68.1 (C-4), 64.5 (C-6), 54.2 (C-2), 20.9 & 20.8 (*Me*COO), 18.4 (C-2'), 11.70 & 11.67 (C-5' & 5").

The deacetylation reaction is anomerically selective for the α-anomer as reported by Fiandor et al.[121] For Ac$_3$GalNCyoc and Ac$_3$GlcNCyoc this can be seen by the presence of a doublet in the ^{1}H NMR spectra with a coupling constant approximately 3 Hz (the β-anomer would have a much larger constant of around 8 Hz as the two protons are antiperiplanar to each other). In the ^{1}H NMR spectrum of Ac$_3$ManNCyoc the anomeric proton was not resolved, but we assume it is also the α-anomer.

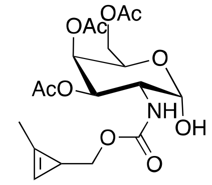

Ac$_3$GalNCyoc: (32% yield) δ_H (400 MHz, CD_3OD) 6.66 (s, 1H), 5.42 (d, *J* = 3.2 Hz, 1H), 5.21 (d, *J* = 3.2 Hz, 1H), 4.47 (t, *J* = 6.4 Hz, 1H), 3.76-4.22 (m, 6H), 2.18 (s, 3H), 2.16 (s, 3H), 2.03 (s, 3H), 1.97 (s, 3H).
δ_C (100 MHz, CD_3OD) 170.8, 170.8, 170.7, 157.8, 120.8, 101.4, 92.1, 72.2, 68.6, 67.8, 65.7, 61.7, 49.9, 19.3, 19.2, 19.2, 16.9, 10.2.

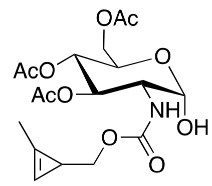

Ac_3GlcNCyoc: (22% yield) δ_H (400 MHz, CD_3OD) 6.66 (s, 1H), 5.29 (t, J = 10.1 Hz, 1H), 5.19 (s, 1H), 5.01 (t, J = 9.6 Hz, 1H), 4.22-4.29 (m, 2H), 4.06-4.15 (m, 1H) 3.82-4.03 (m, 3H), 2.14 (s, 3H), 2.07 (s, 3H), 2.03 (s, 3H), 2.00 (s, 3H), 1.63 (t, J = 5.3 Hz, 1H).
δ_C (100 MHz, CD_3OD) 171.1, 170.8, 170.0, 157.5, 120.6, 101.4, 91.6, 71.2, 69.2, 66.9, 65.5, 62.2, 54.1, 19.4, 19.3, 19.3, 16.8, 10.8.

HRMS: (ESI+) Ac_3ManNCyoc: 438.1377, Ac_3GalNCyoc: 438.1377, Ac_3GlcNCyoc: 438.1375 ($C_{18}H_{25}NO_{10}.Na^+$, requires 438.1376).

Tetrazine-PEG_{11}-AlexaFluor647[4] and TMDBIO-Lys- AlexaFluor647[5] were synthesised as previously described.

1.3 *In Vitro* Experiments

Cell surface glycan labelling and detection by flow cytometry

In Costar® clear TC-treated 6-well plates, cells were seeded at a density of 2.5 x 10^5 cells per well, in 3 ml of medium. After 24 h the cells were pulsed with the sugar (125 µM) or left unpulsed. After 24 h the medium was placed into a centrifuge tube, the flasks and cells were washed with PBS (phosphate buffered saline; water, NaCl, KCl, Na_2HPO_4, KH_2PO_4; Fisher Scientific, Loughborough, UK). Each well was treated with Gibco 0.25% Trypsin- 1 mM EDTA (0.5 mL) per well and returned to the incubator for 3 minutes to facilitate dissociation of the cells from the vessel. The trypsin was quenched by addition of cell medium (1.5 mL) and the contents of each well were transferred to the centrifuge tube and centrifuged (1000 g, 4 °C, 3 min). The cells were resuspended in cold FACS buffer (1% FBS in PBS), transferred to 1.5 mL Eppendorf tubes, centrifuged and resuspended in 100 µL labelling buffer (5 µM Tz-Alexa647 or TMDIBO-Lys-Alexa647 + 50 nM Sytox green cell death stain). The Eppendorf tubes were incubated in a hot block with orbital shaking (450 rpm, 37 °C, 1 h). The cells were centrifuged, washed three times with 700 µL ice-cold FACS buffer, filtered through a 50 µm cut-off membrane into flow cytometry tubes, and kept on ice. Each sample was analysed by flow cytometry (model S6 Symphony, BD Oxford, UK) using 10,000 events. Data analysis was performed using FlowJo flow cytometry analysis software (Tree Star, Ashland, OR). The viable cell population (population of interest) was determined by gating cells to exclude those with high levels of SYTOX Green (cell death marker). The far-red median fluorescence intensity (MFI, Alexa Fluor 647 fluorophore) of the viable cell population was then assessed. Data points were collected in triplicate.

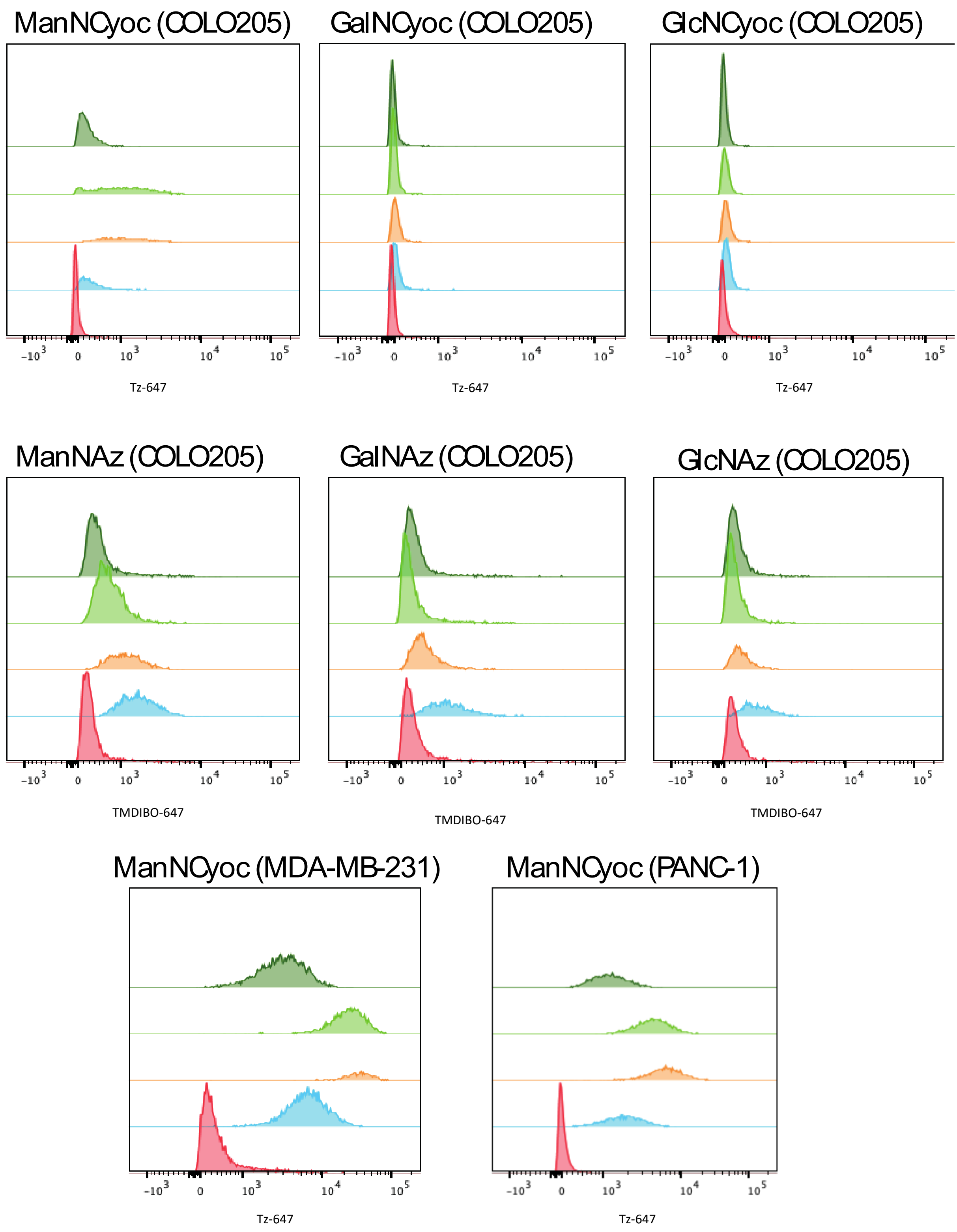

Figure S1.1 - Flow cytometry results with the different sugars and cell lines. Histograms giving number of events (vertical axis) versus fluorescence intensity (horizontal axis). In each panel the sugars used, from top to bottom, are mono-, di-, tri- and tetra-acetylated and then the no-sugar control.

Fluorescent microscopy of cell surface labelled glycans

PANC-1 cells seeded into an Ibidi µ-Slide 8 well plate at $1{\times}10^5$ cells ml^{-1} and allowed to adhere to the plate surface for 24 h. After adhesion, cells were pulsed with either Ac_3ManCyoc (125 µM) (+), or unpulsed (-) and left for 24 h. Cells were then washed 3 times in ice cold FACS buffer and incubated (60 min, 37 °C) in situ in 150 µL of FACS buffer containing 5 µM Tz-PEG_{11}-AlexaFluor647. After being washed, as above, cells were fixed in PBS containing 4% formalin (15 min, 37 °C), washed again as above, then stained with 150 µL of 300 nM DAPI (15 min, 37 °C), then washed as above, and 300 µL PBS was added. Slides were scanned on a high content imaging Operetta CLS (Perkin Elmer) microscope in confocal mode using a sCMOS camera and a 40x water immersion objective. Images were taken in two channels, DAPI and AlexaFluor647 and in 3D with z stacks at intervals of 0.5 µm. LED power and exposure times remained the same between experimental and control conditions within each experiment. Images were processed and exported from Harmony software (Perkin Elmer) then Fiji (ImageJ) software was used to create the plot profile of AlexaFluor 647 intensity distribution across the cells.

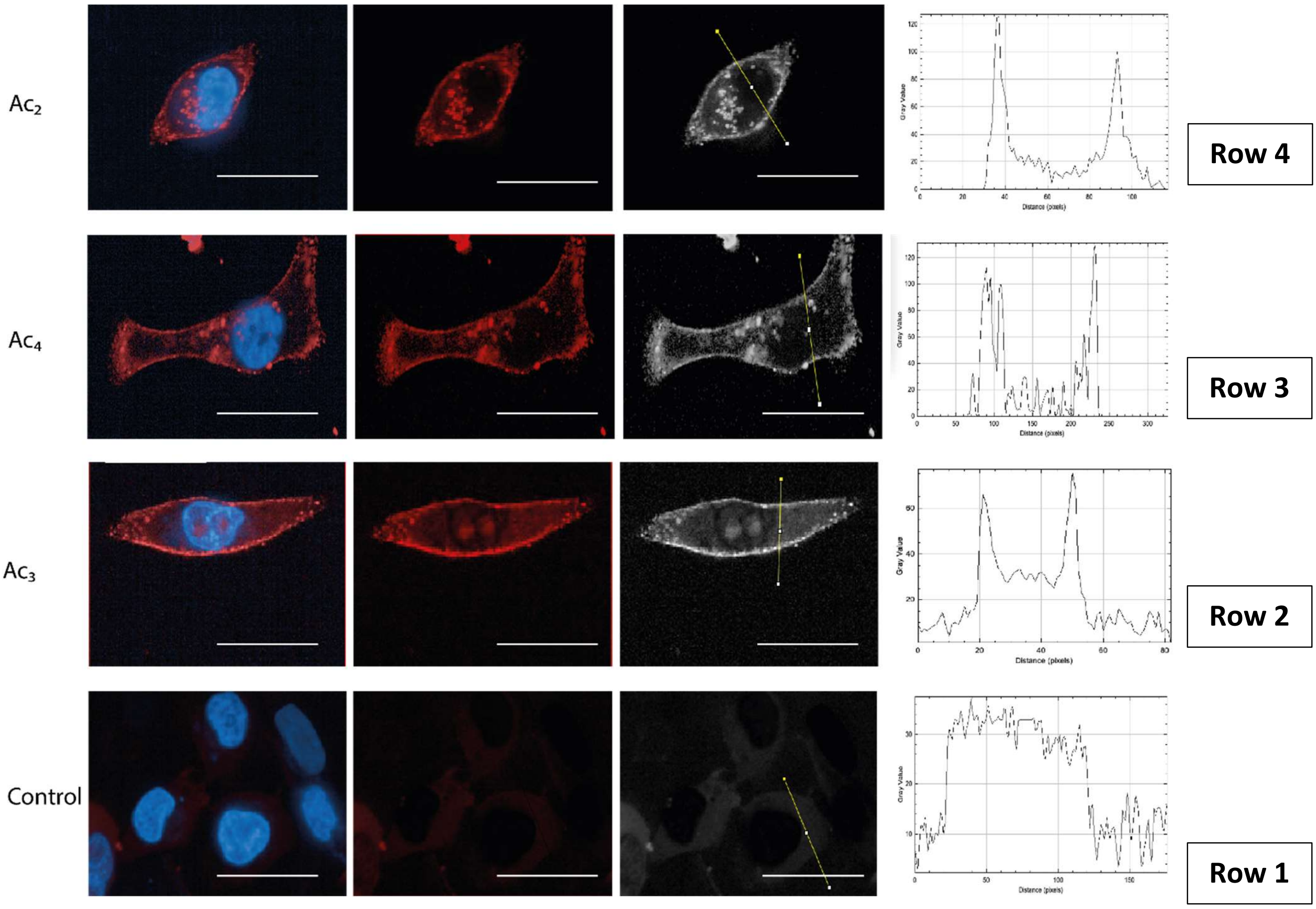

Figure S1.2 - Confocal microscopy images of MDA-MB-2321 cells after the treatment described in the preceding paragraph. Column 1: overlay of DAPI and AlexaFluor 647 channels. Column 2: AlexaFluor 647 channel. Column 3: AlexaFluor 647 channel showing the cross-section of the cell analysed. Column 4: Fluorescence intensity along the cross-section line shown in row 3, showing that the fluorescence caused by the tagged sugars is concentrated at the cell surface, whereas the background fluorescence in the no-sugar control is uniform across the cell.

The effect of Ac$_3$ManNCyoc treatment on cell viability and growth

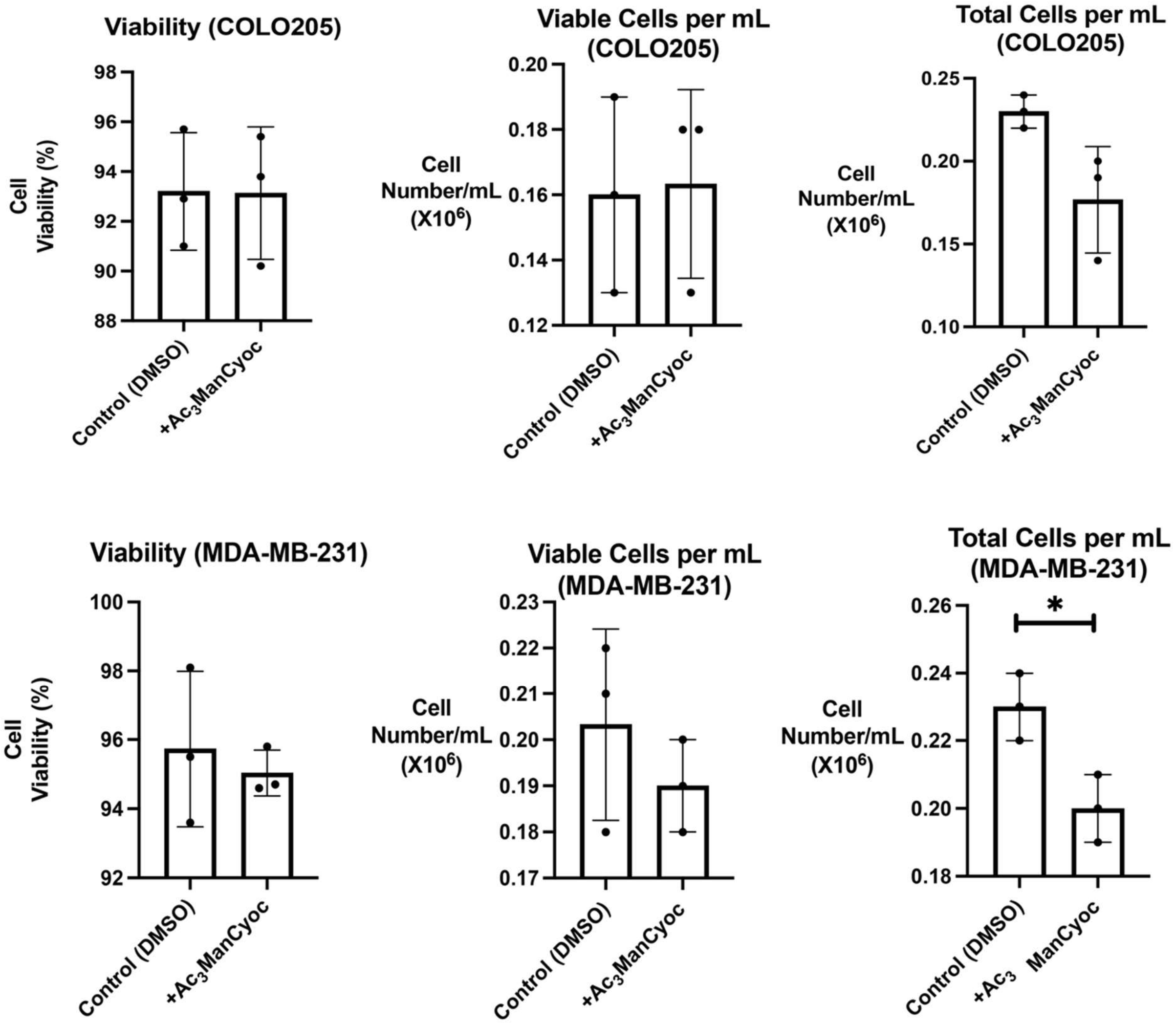

Figure S1.3 - The effect of dual sugar treatment on the viability and growth of COLO205 and MDA-MB-231 cells. cells were incubated in the presence of Ac$_3$ManCyoc (125 µM) or treated with the same volume of DMSO alone for 24 h. The cells pellet was collected and resuspended before being analysed on a Vi-CELL XR cell viability analyser. n=3 technical replicates. Statistical analysis was performed using an unpaired t test with Welch correction (**** $P \leq 0.0001$,*** $P \leq 0.001$,** $P \leq 0.01$,* $P \leq 0.05$).

1.4 References

1 S. T. Laughlin and C. R. Bertozzi, *Nat. Protoc.*, 2007, **2**, 2930–2944.
2 D. M. Patterson, K. A. Jones and J. A. Prescher, *Mol. BioSyst*, 2014, **10**, 1693–1697.
3 J. Fiandor, M. T. Garciá-López, F. G. De Las Heras and P. P. Méndez-Castrillón, *Synth.*, 1985, **1985**, 1121–1123.
4 H. Stöckmann, A. A. Neves, H. A. Day, S. Stairs, K. M. Brindle and F. J. Leeper, *Chem. Commun.*, 2011, **47**, 7203–7205.
5 A. A. Neves, H. Stöckmann, Y. A. Wainman, J. C.-H. Kuo, S. Fawcett, F. J. Leeper and K. M. Brindle, *Bioconjug. Chem.*, 2013, **24**, 934–941.

2. ^{1}H and ^{13}C NMR Spectra

2.1 1D Spectra

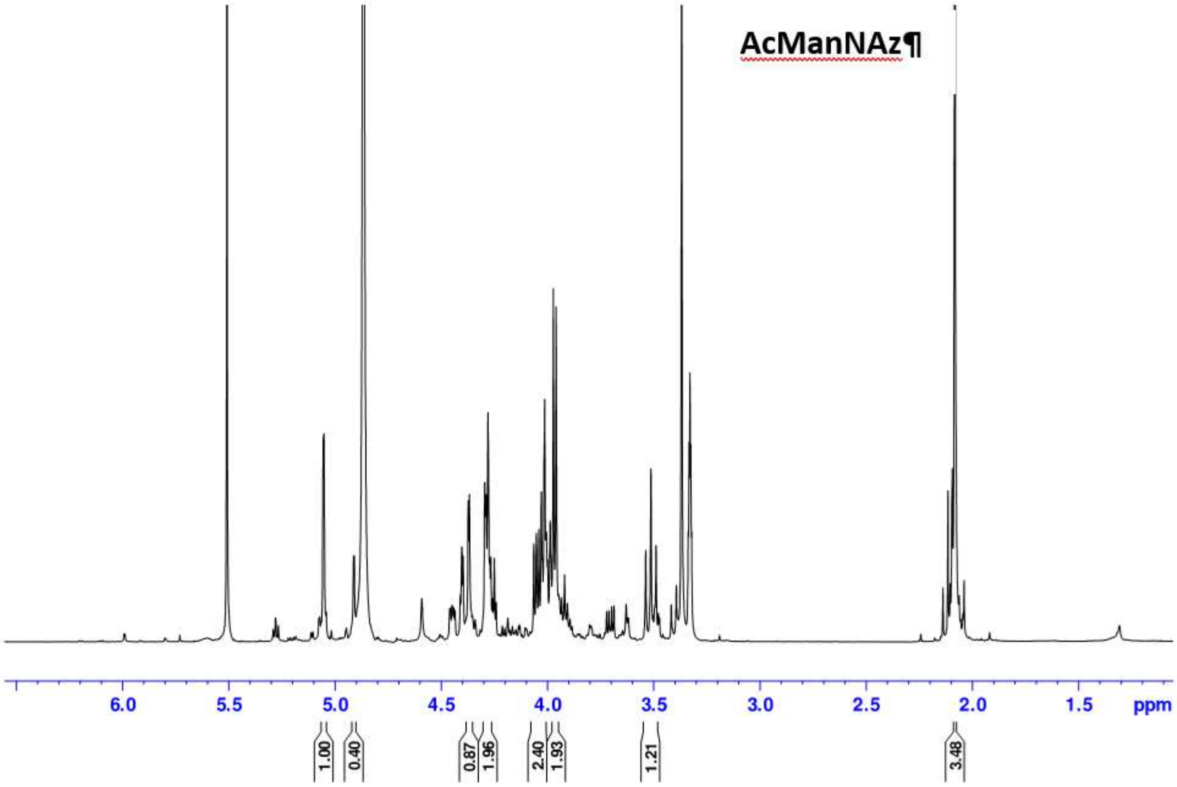

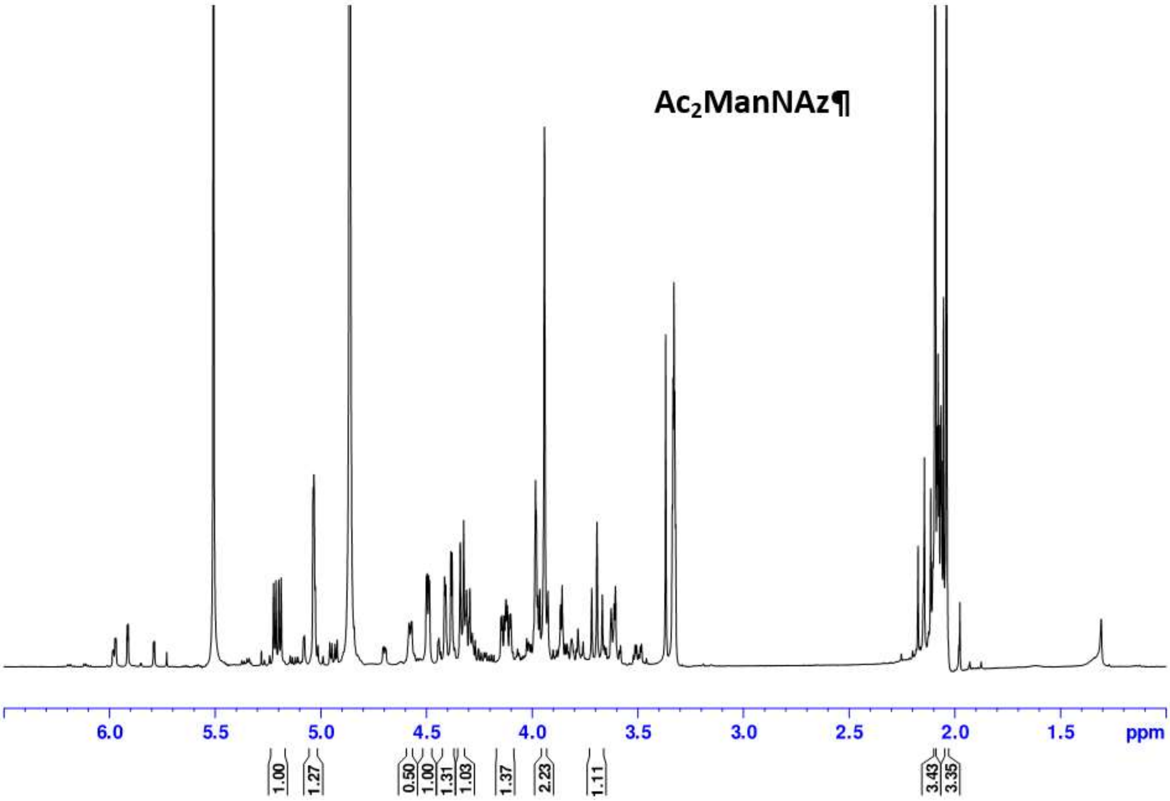

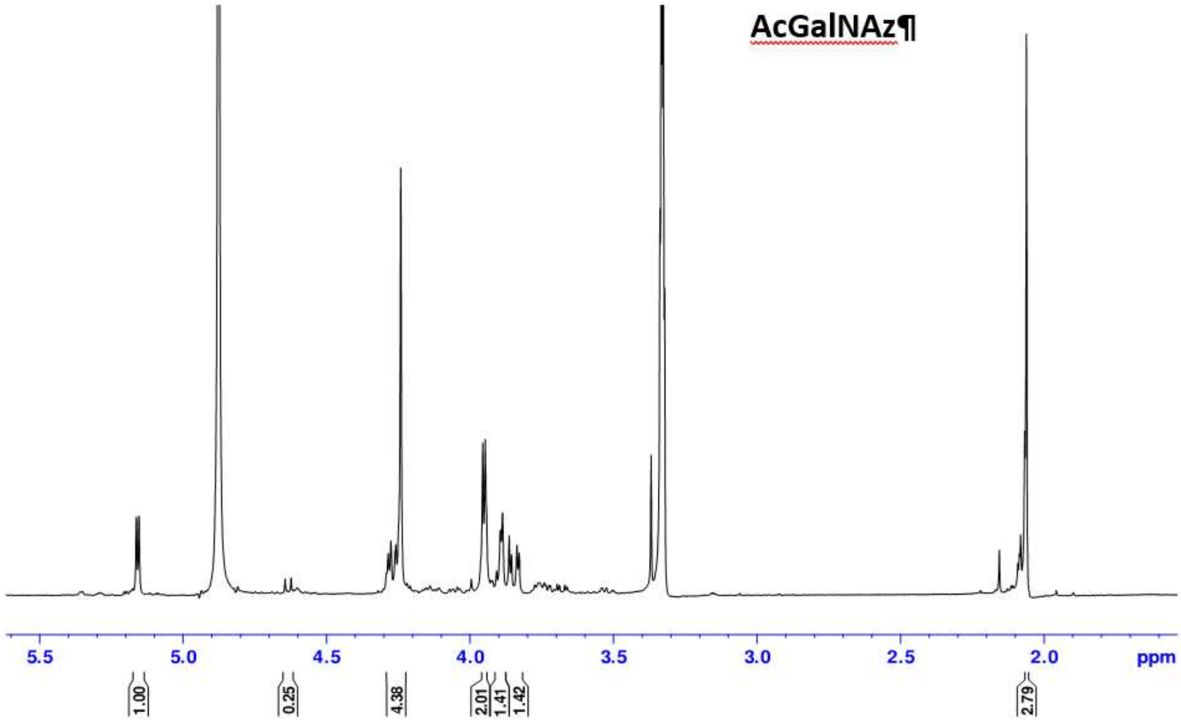
AcGalNAz¶
5.5
5.0
4.5
4.0
3.5
3.0
2.5
2.0
ppm
1.00
0.25
4.38
2.01
1.41
1.42
2.79

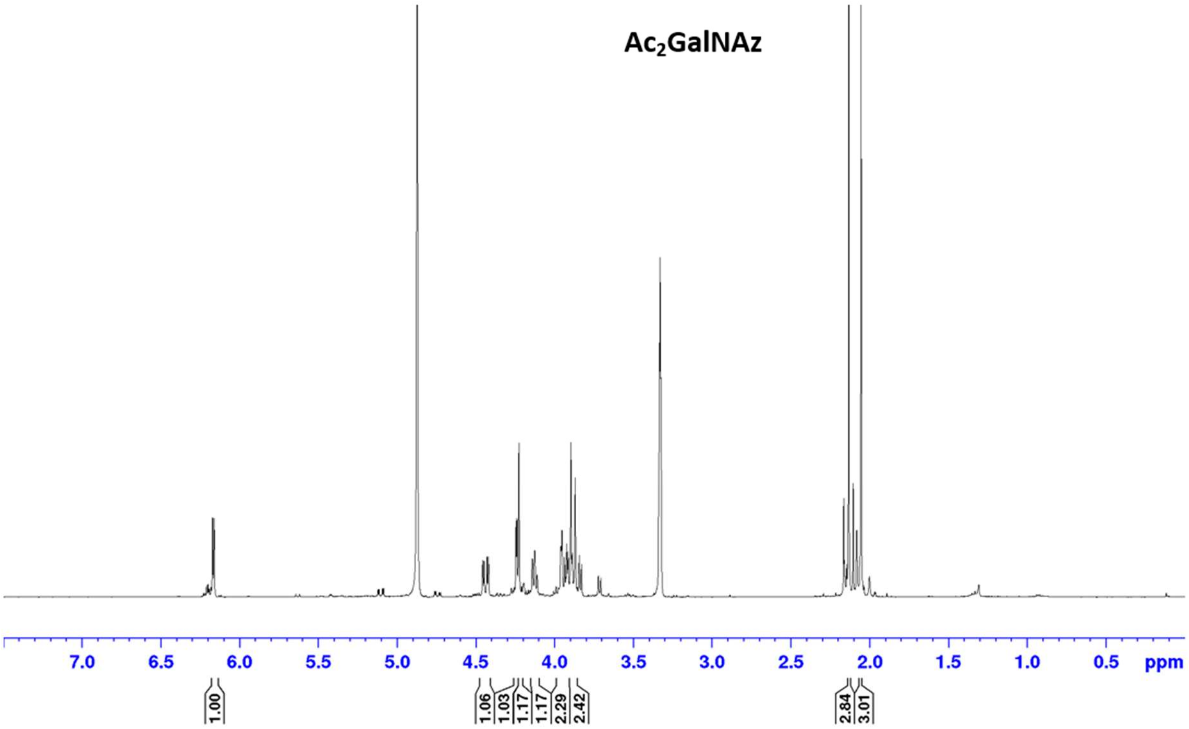
Ac₂GalNAz
7.0
6.5
6.0
5.5
5.0
4.5
4.0
3.5
3.0
2.5
2.0
1.5
1.0
0.5
ppm
1.00
1.06
1.03
1.17
1.17
2.29
2.42
2.84
3.01

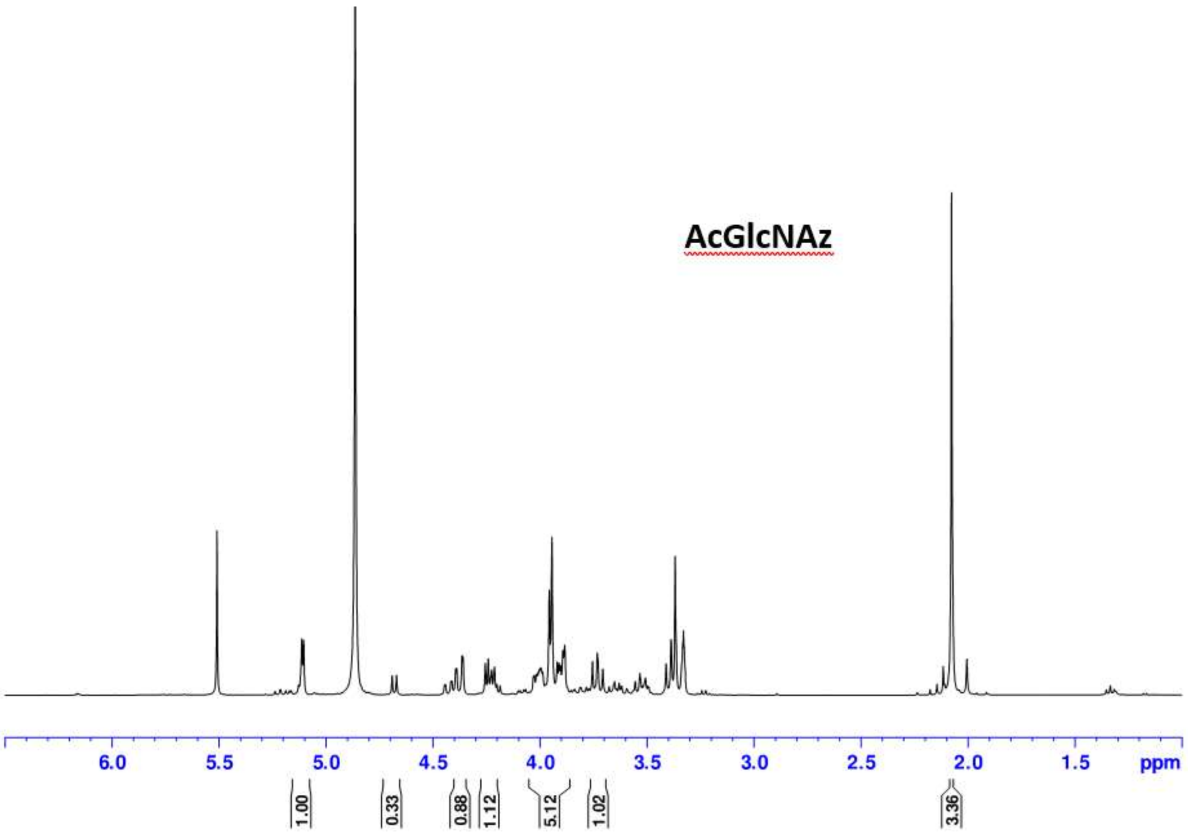
AcGlcNAz
6.0 5.5 5.0 4.5 4.0 3.5 3.0 2.5 2.0 1.5 ppm
1.00
0.33
0.88
1.12
5.12
1.02
3.36

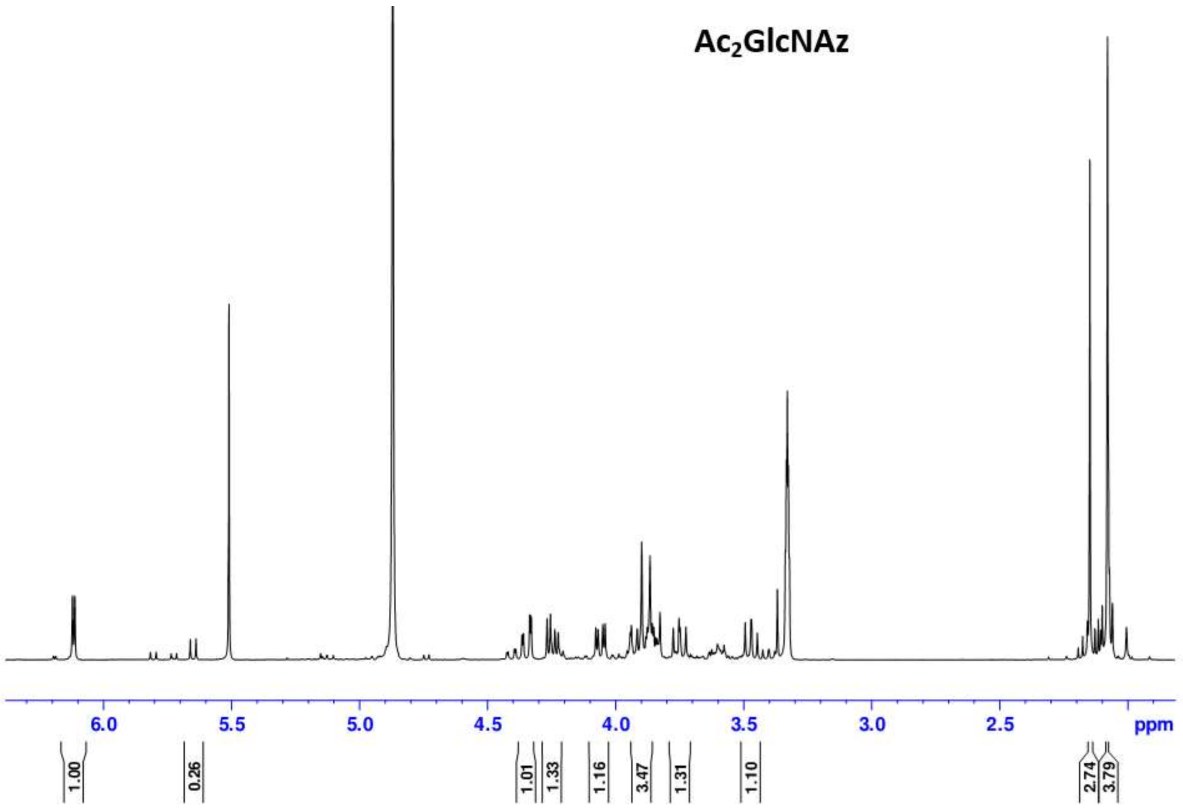
Ac$_2$GlcNAz
6.0 5.5 5.0 4.5 4.0 3.5 3.0 2.5 ppm
1.00
0.26
1.01
1.33
1.16
3.47
1.31
1.10
2.74
3.79

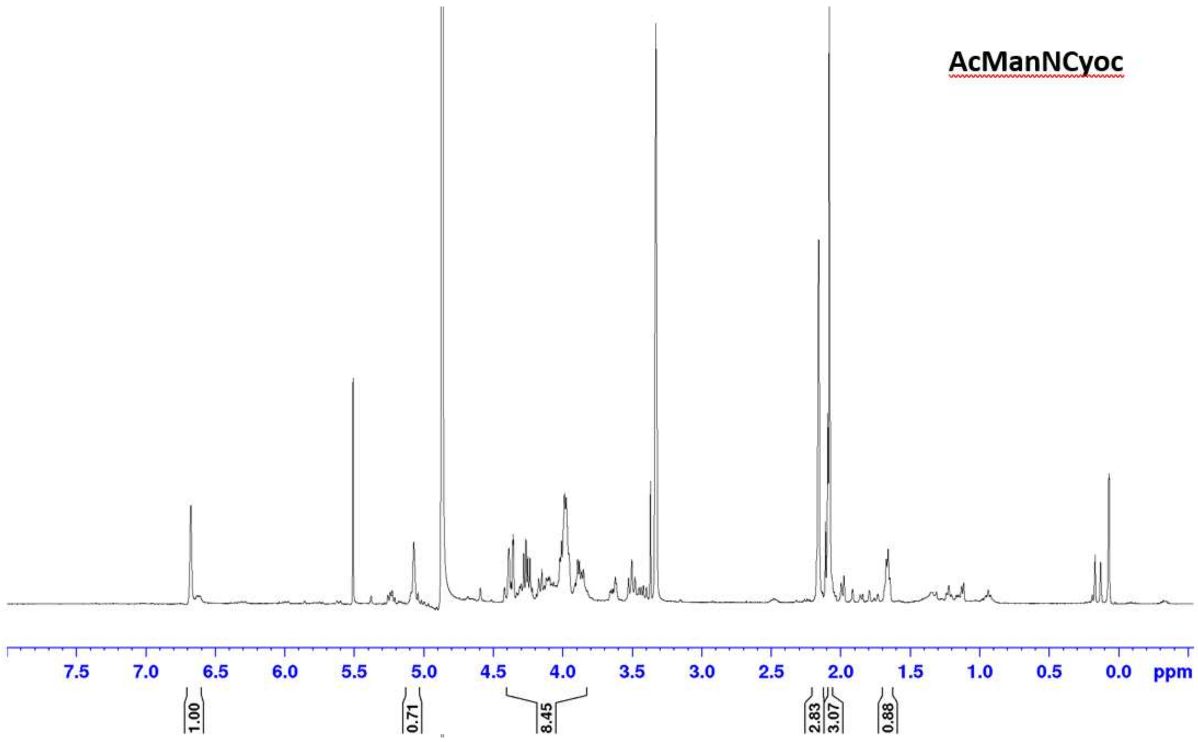
AcManNCyoc
7.5 7.0 6.5 6.0 5.5 5.0 4.5 4.0 3.5 3.0 2.5 2.0 1.5 1.0 0.5 0.0 ppm
1.00
0.71
8.45
2.83
3.07
0.88

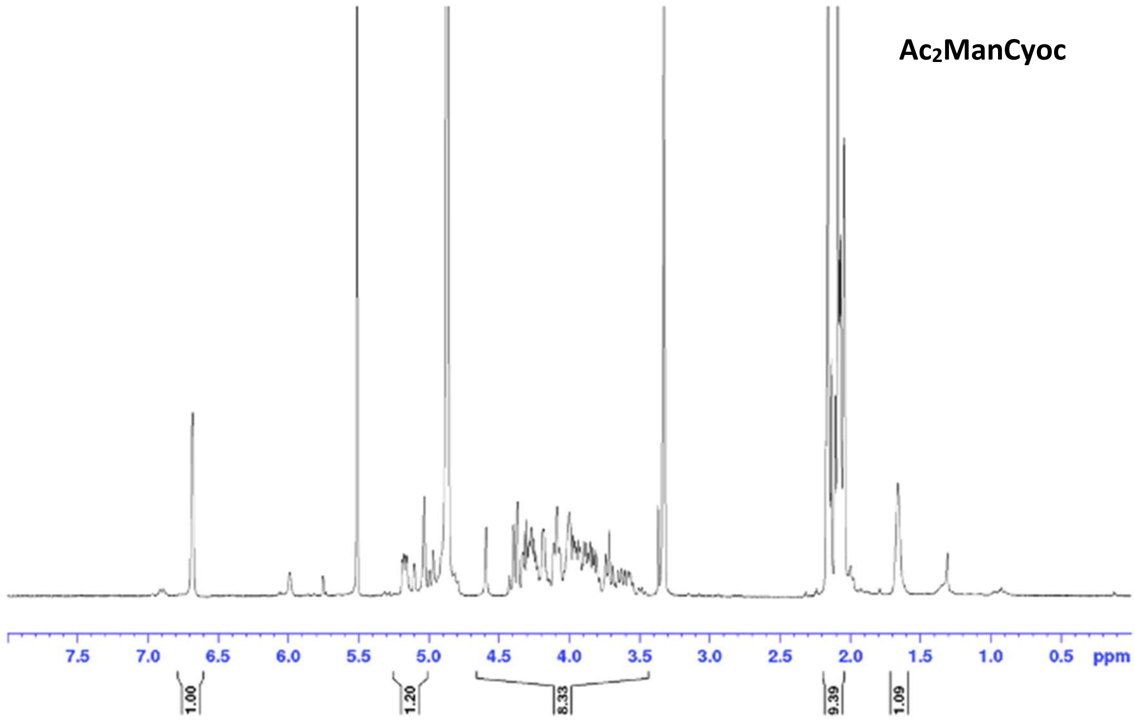
Ac$_2$ManCyoc
7.5 7.0 6.5 6.0 5.5 5.0 4.5 4.0 3.5 3.0 2.5 2.0 1.5 1.0 0.5 ppm
1.00
1.20
8.33
9.39
1.09

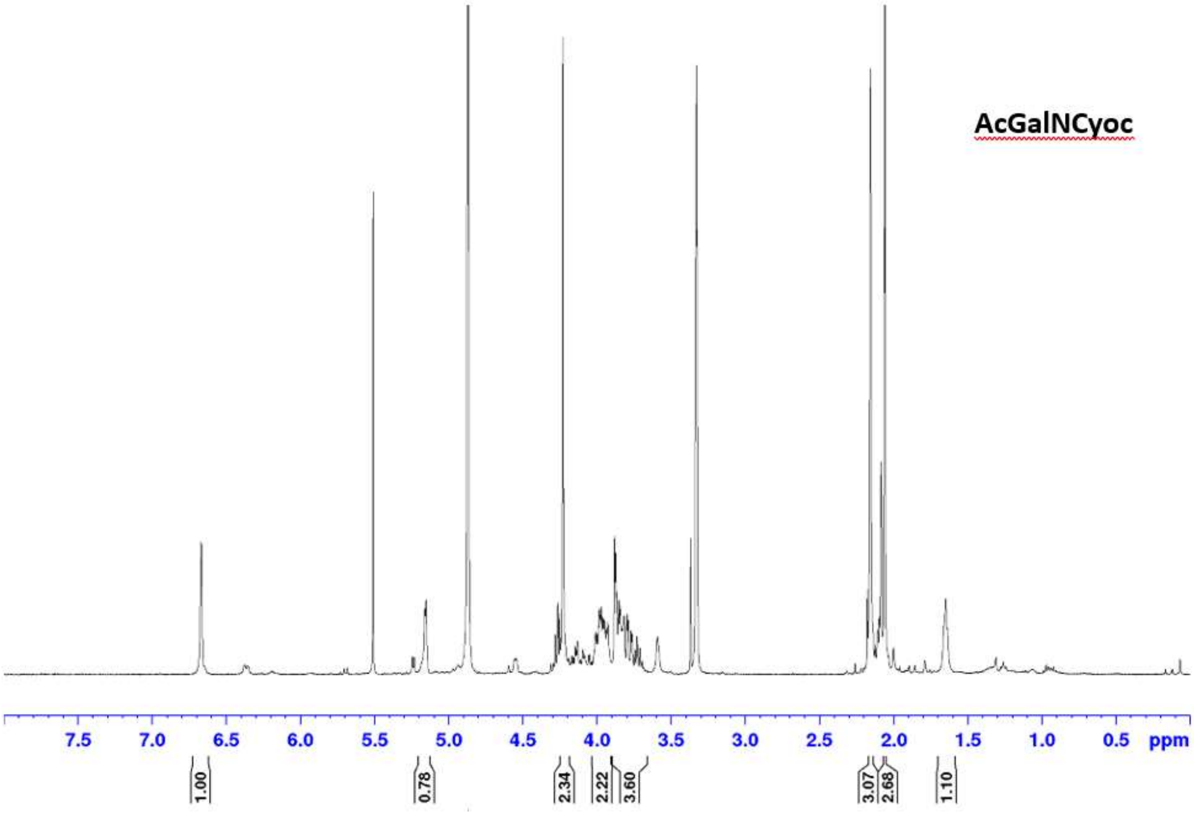
AcGalNCyoc
7.5
7.0
6.5
6.0
5.5
5.0
4.5
4.0
3.5
3.0
2.5
2.0
1.5
1.0
0.5
ppm
1.00
0.78
2.34
2.22
3.60
3.07
2.68
1.10

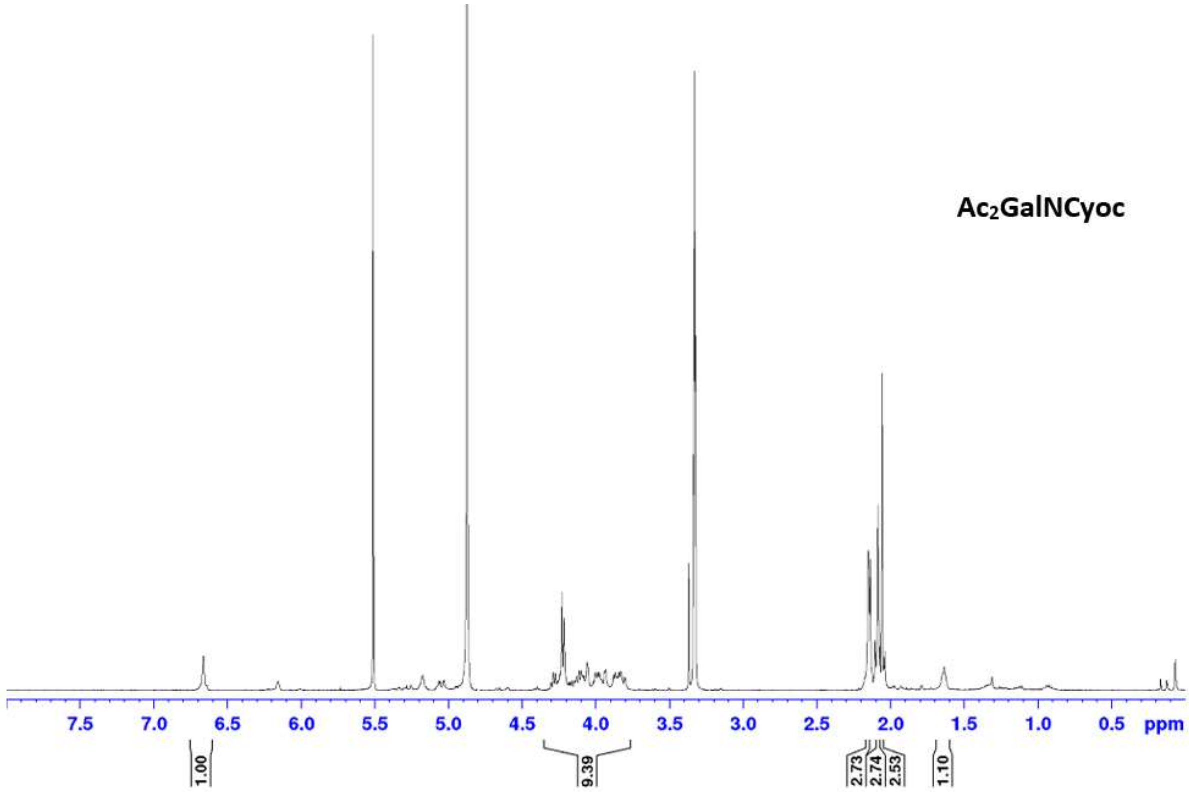
Ac2GalNCyoc
7.5
7.0
6.5
6.0
5.5
5.0
4.5
4.0
3.5
3.0
2.5
2.0
1.5
1.0
0.5
ppm
1.00
9.39
2.73
2.74
2.53
1.10

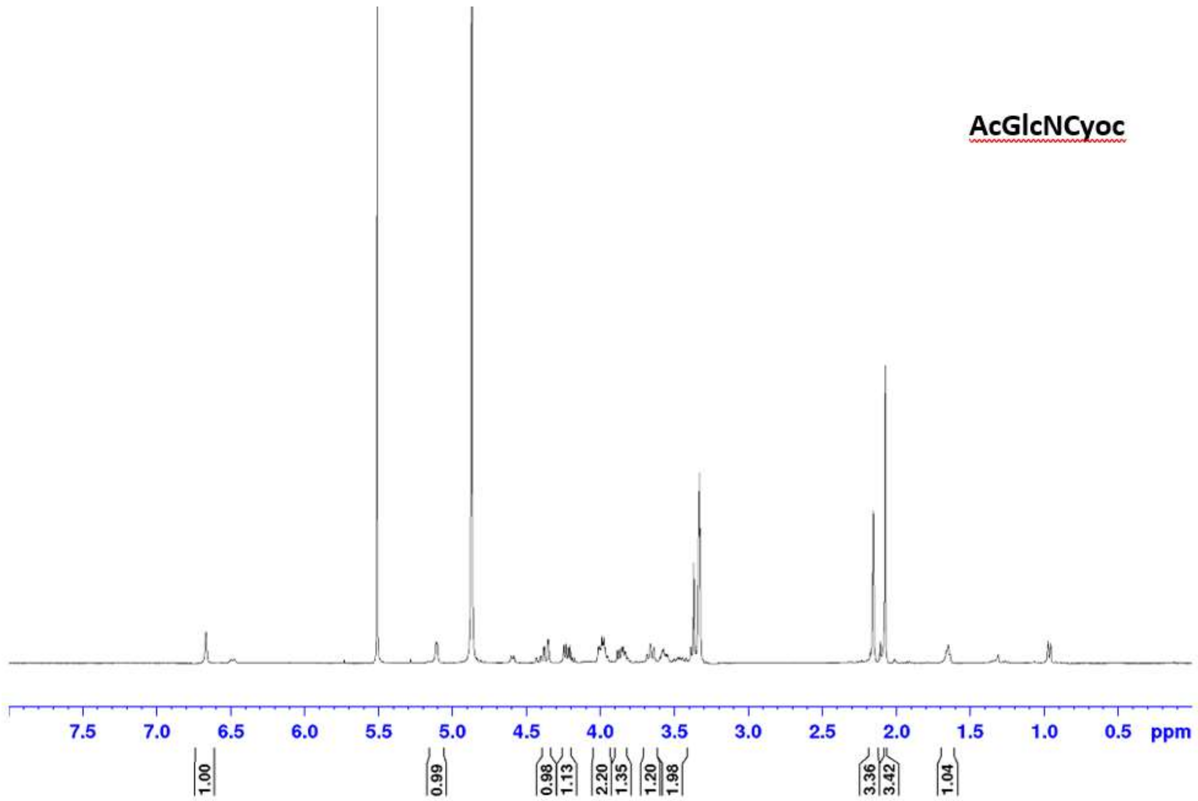
AcGlcNCyoc
ppm

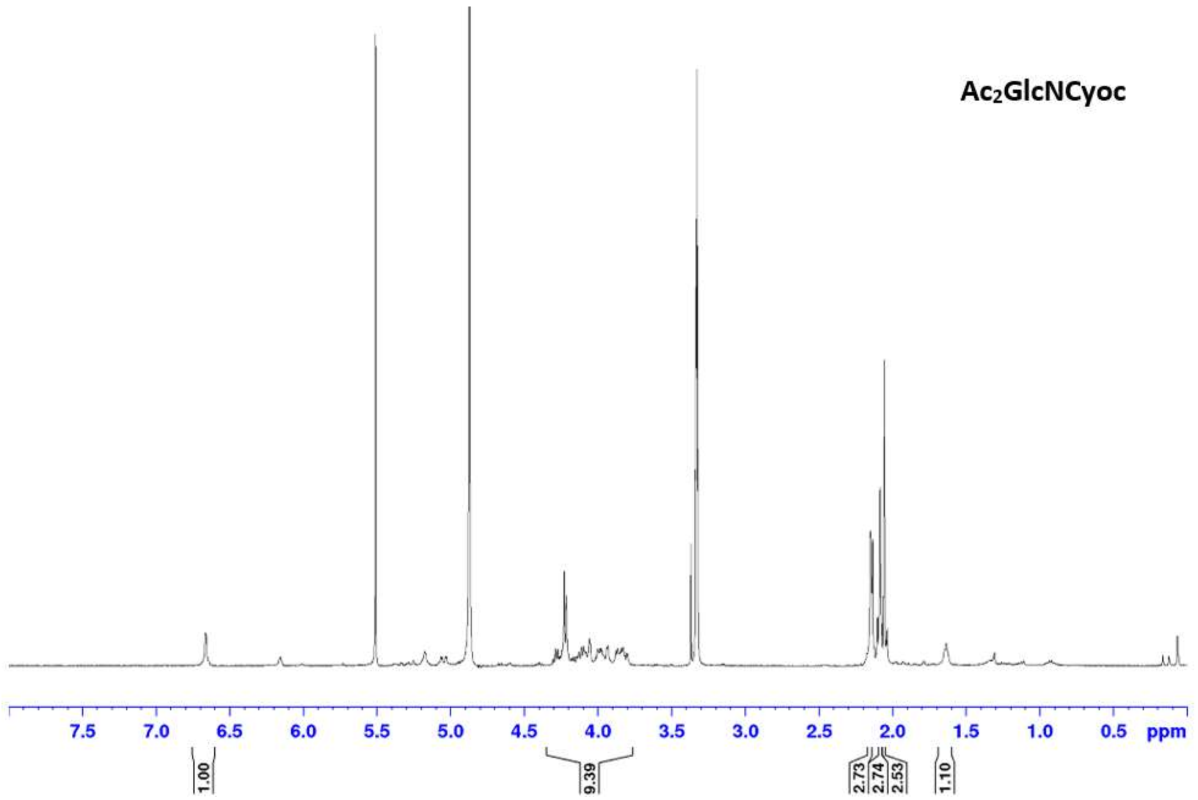
Ac$_2$GlcNCyoc
ppm

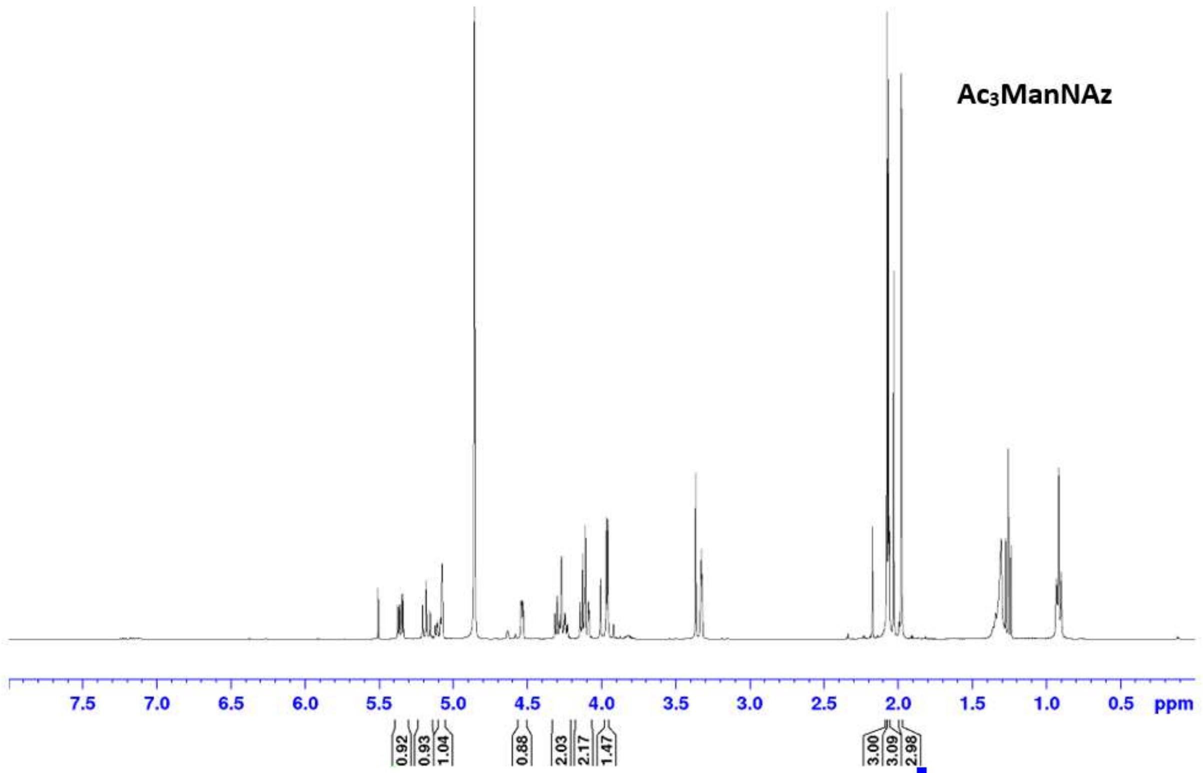
Ac3ManNAz
7.5 7.0 6.5 6.0 5.5 5.0 4.5 4.0 3.5 3.0 2.5 2.0 1.5 1.0 0.5 ppm
0.92 0.93 1.04 0.88 2.03 2.17 1.47 3.00 3.09 2.98

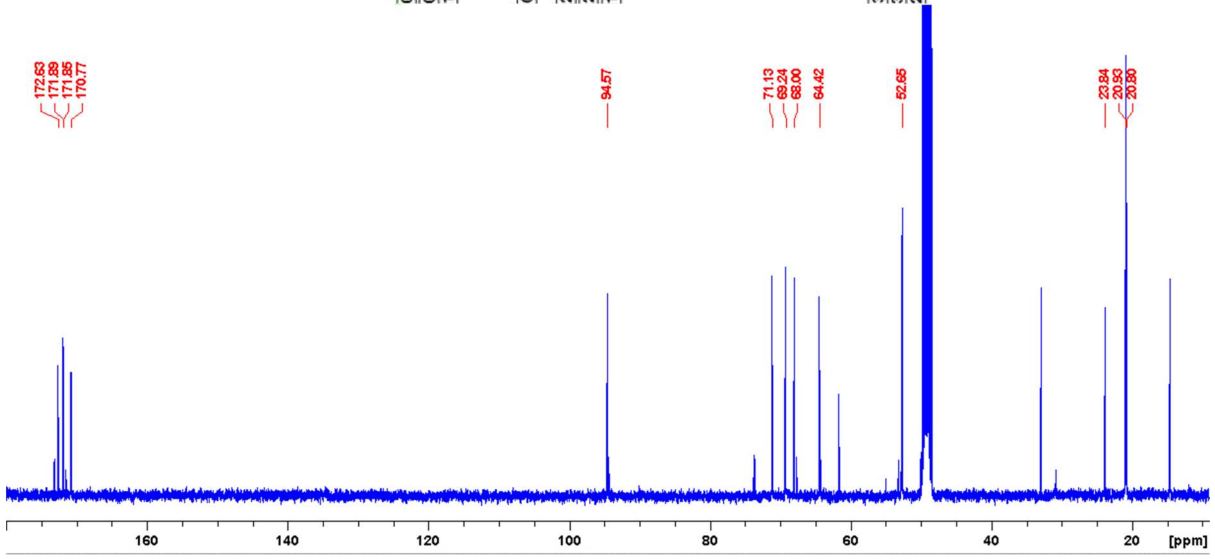
172.63 171.89 171.85 170.77
94.57
71.13 69.24 68.00 64.42 52.65
23.94 20.93 20.80
160 140 120 100 80 60 40 20 [ppm]

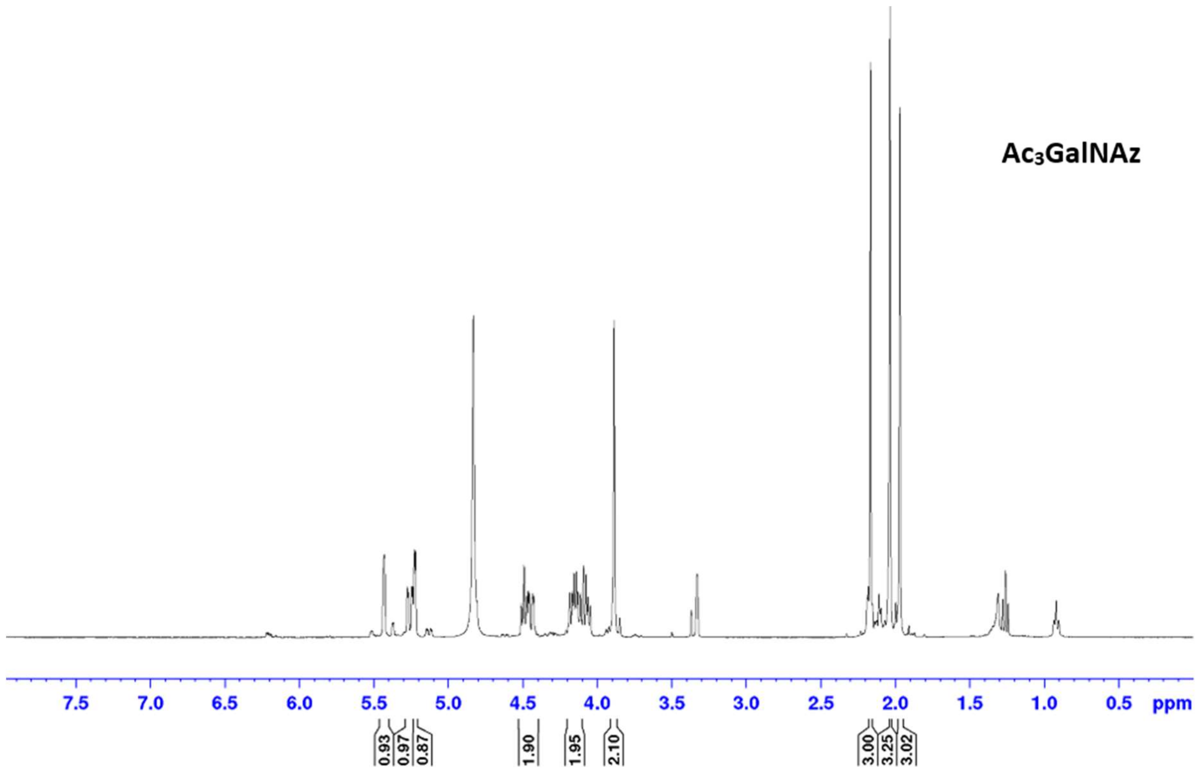
Ac$_3$GalNAz
7.5 7.0 6.5 6.0 5.5 5.0 4.5 4.0 3.5 3.0 2.5 2.0 1.5 1.0 0.5 ppm
0.93 0.97 0.87 1.90 1.95 2.10 3.00 3.25 3.02

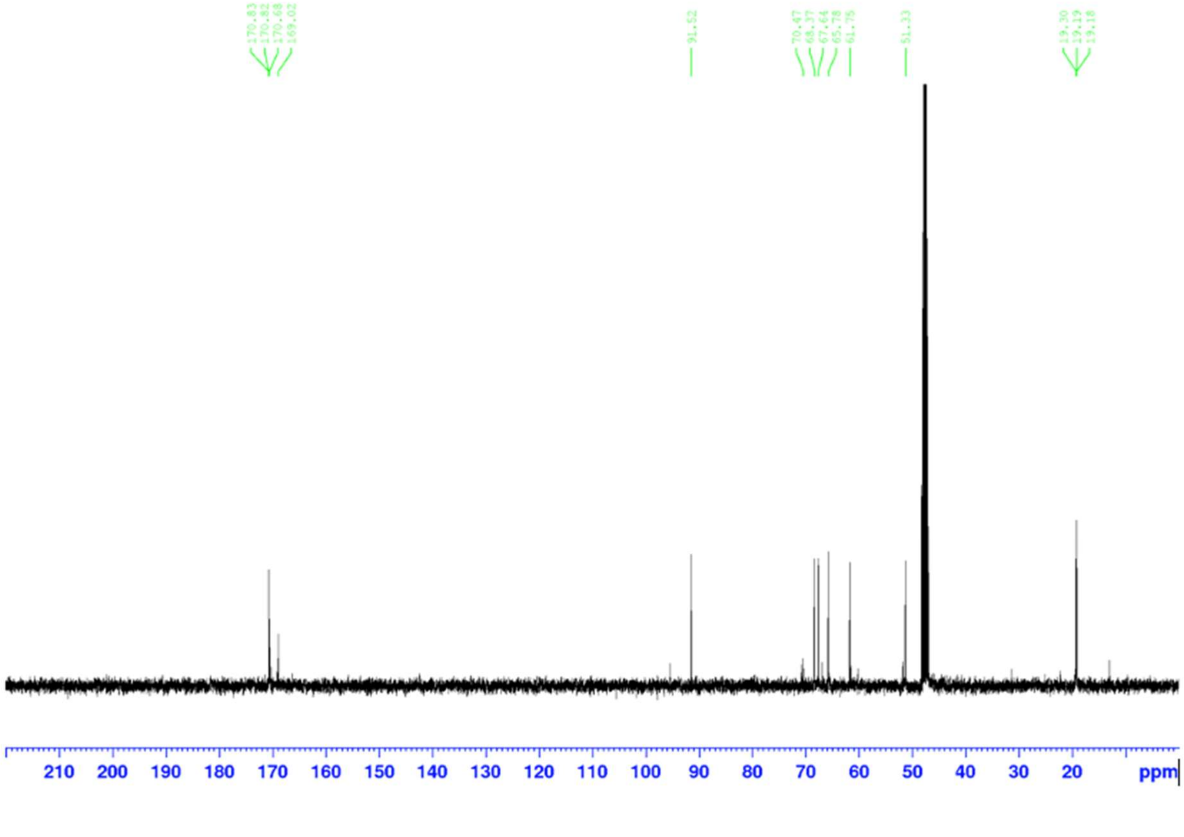
210 200 190 180 170 160 150 140 130 120 110 100 90 80 70 60 50 40 30 20 ppm

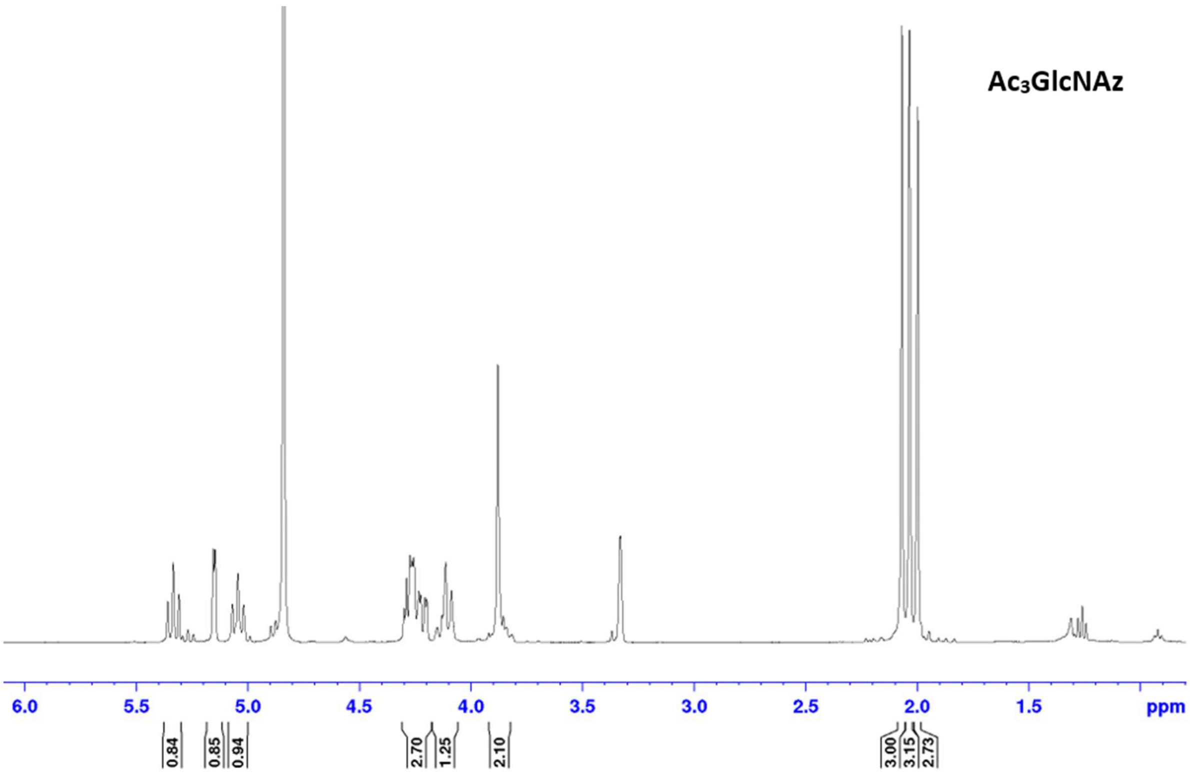
Ac3GlcNAz
6.0
5.5
5.0
4.5
4.0
3.5
3.0
2.5
2.0
1.5
ppm
0.84
0.85
0.94
2.70
1.25
2.10
3.00
3.15
2.73

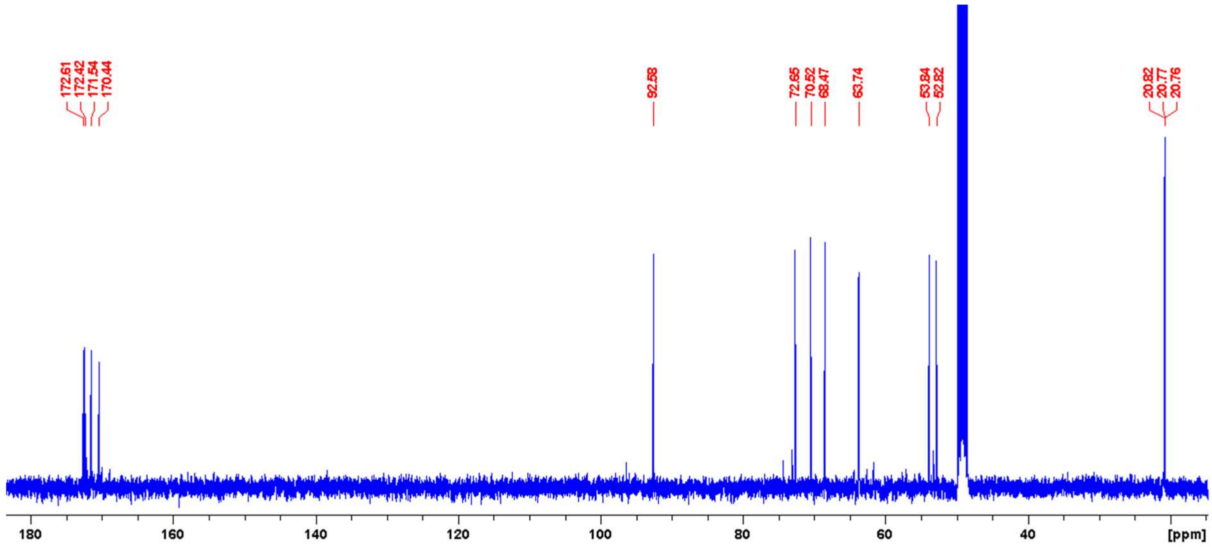
172.61
172.42
171.54
170.44
92.58
72.65
70.52
68.47
63.74
53.94
52.82
20.82
20.77
20.76
180
160
140
120
100
80
60
40
[ppm]

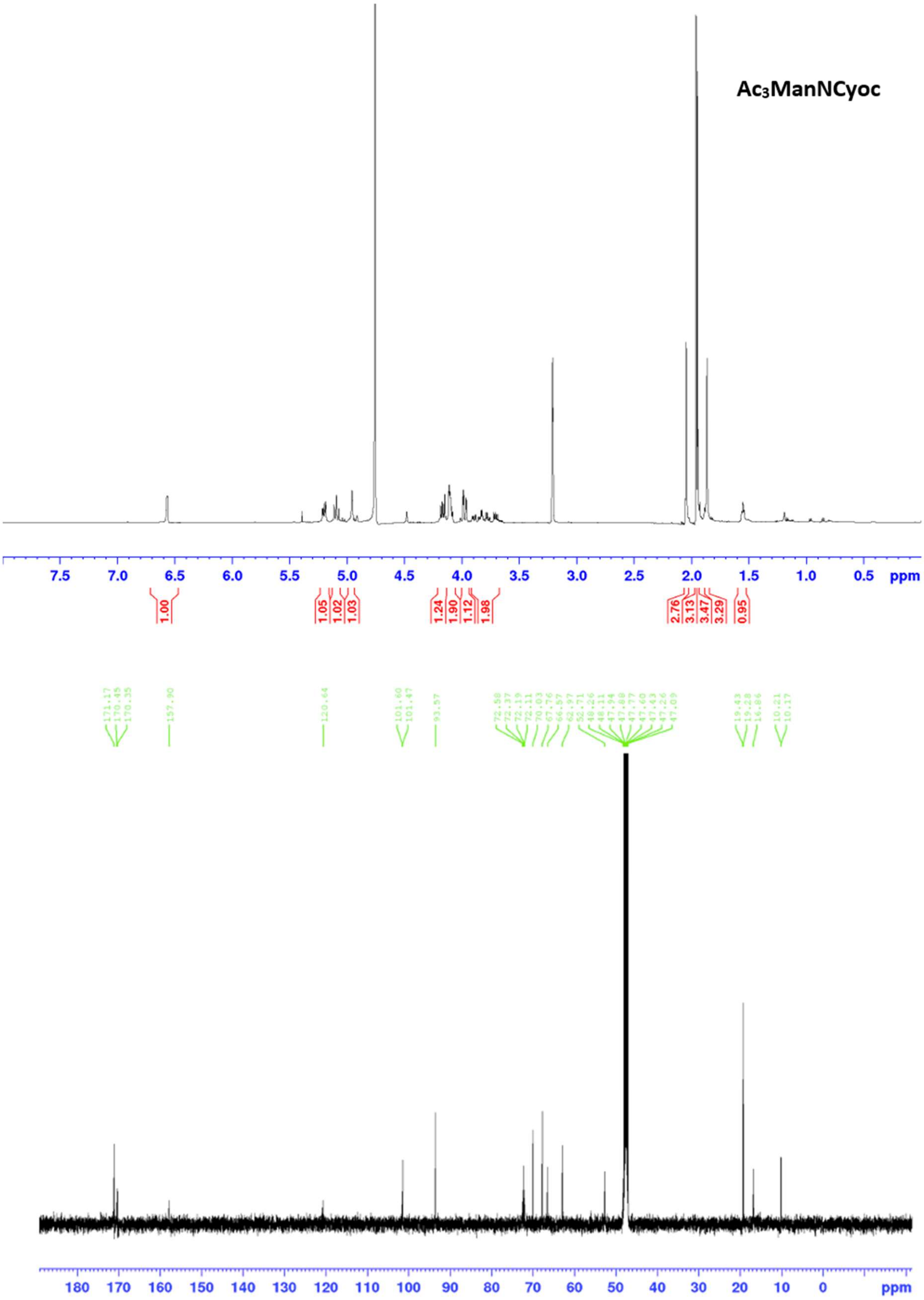
Ac$_3$ManNCyoc
ppm
ppm

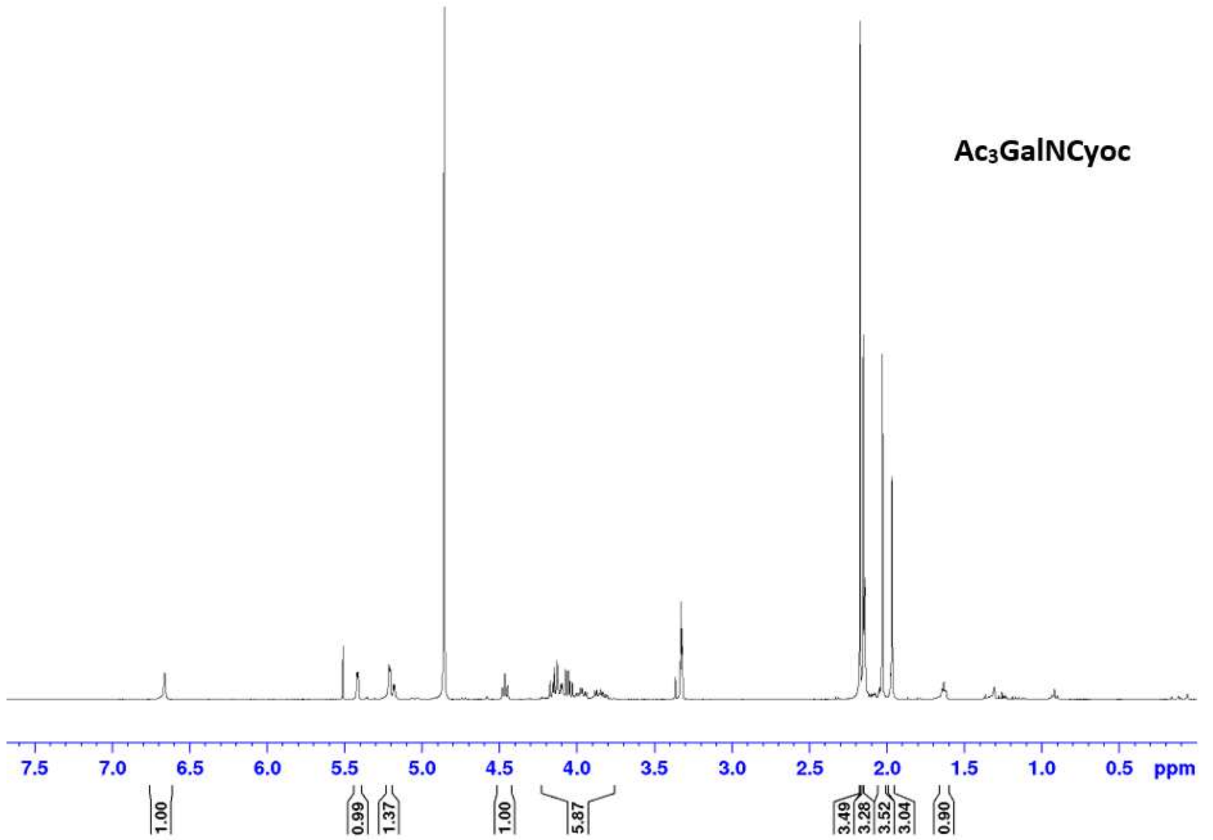
Ac3GalNCyoc
7.5 7.0 6.5 6.0 5.5 5.0 4.5 4.0 3.5 3.0 2.5 2.0 1.5 1.0 0.5 ppm
1.00 0.99 1.37 1.00 5.87 3.49 3.28 3.52 3.04 0.90

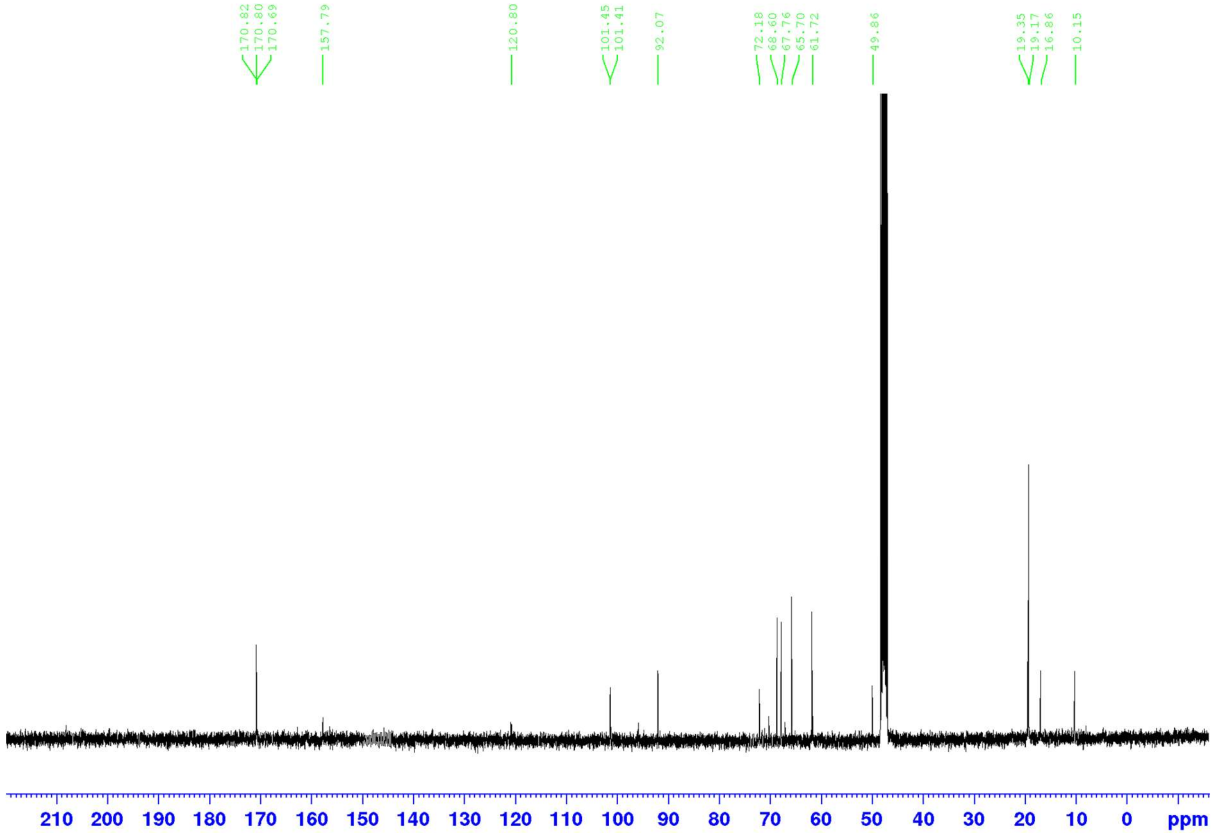
170.82 170.80 170.69 157.79 120.80 101.45 101.41 92.07 72.18 68.60 67.76 65.70 61.72 49.86 19.35 19.17 16.86 10.15
210 200 190 180 170 160 150 140 130 120 110 100 90 80 70 60 50 40 30 20 10 0 ppm

Ac$_3$GlcNCyoc

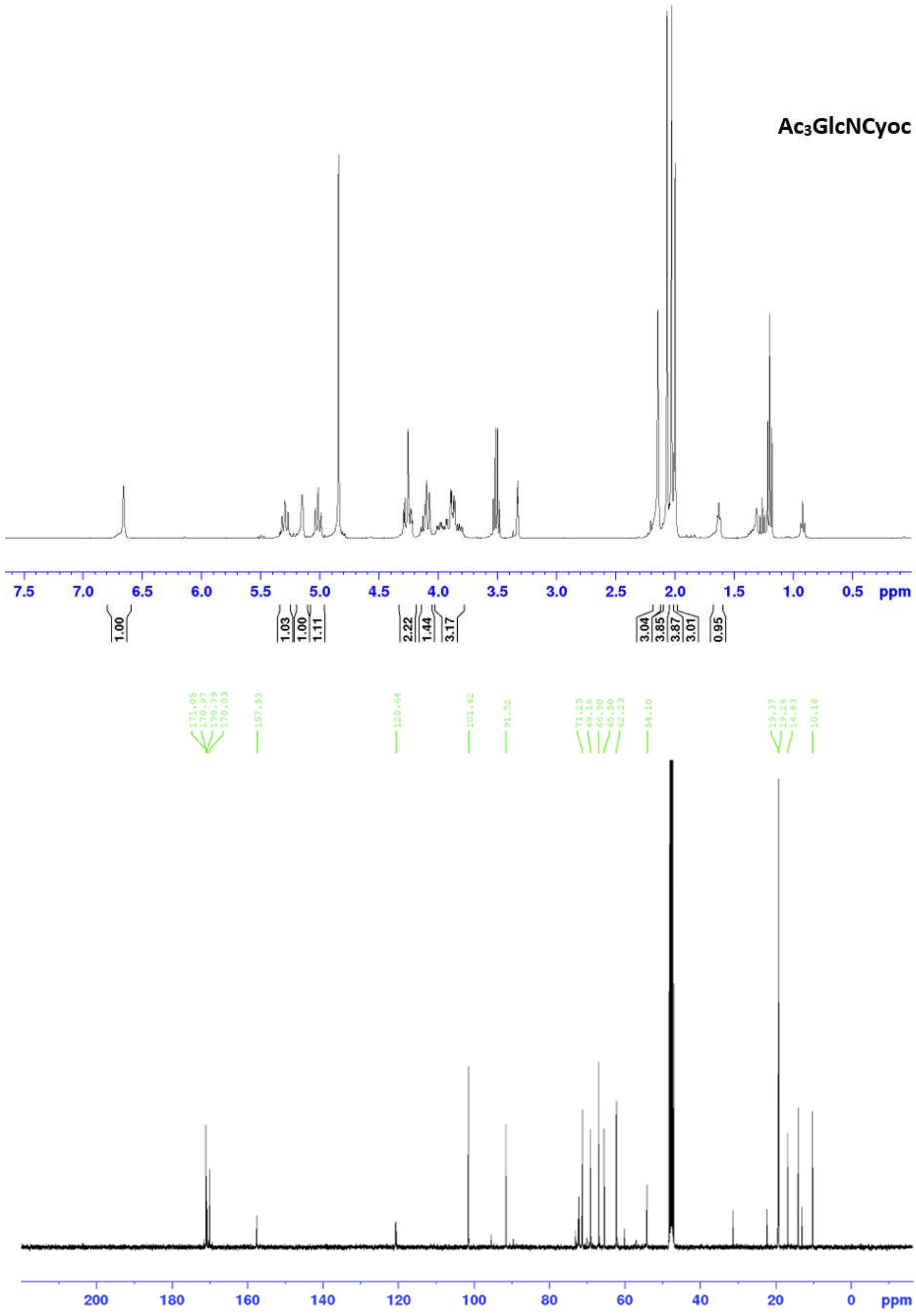

2.2 2D Spectra (Ac$_3$ManNCyoc)

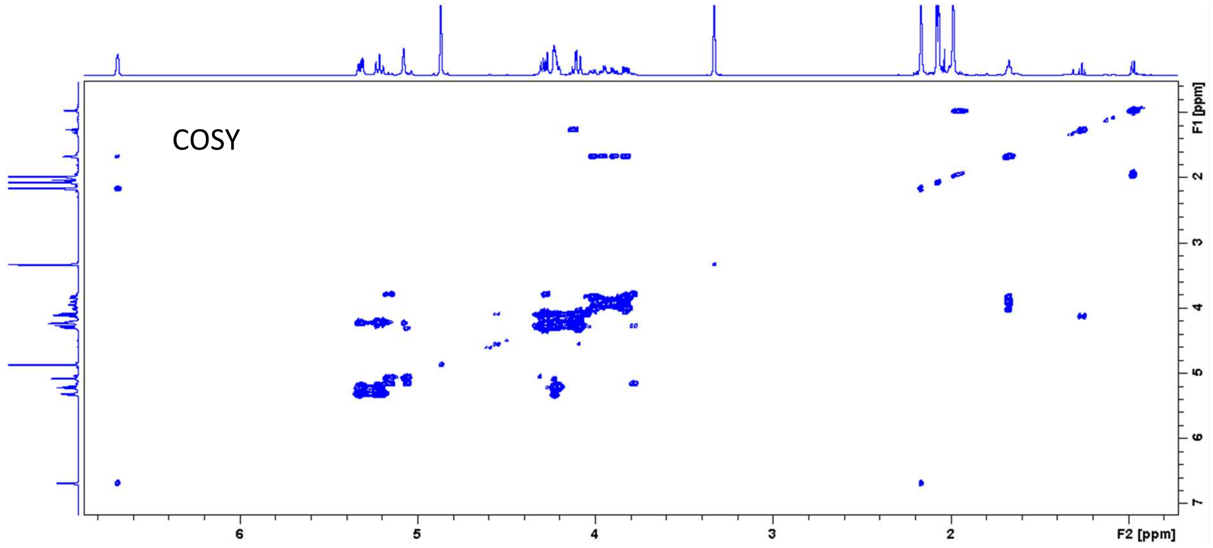

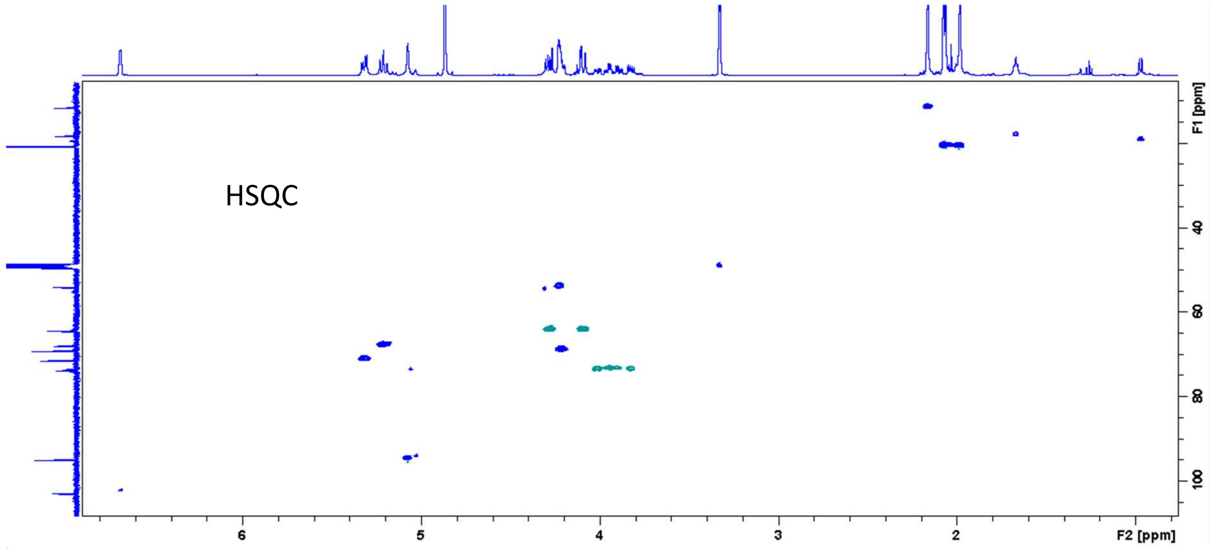

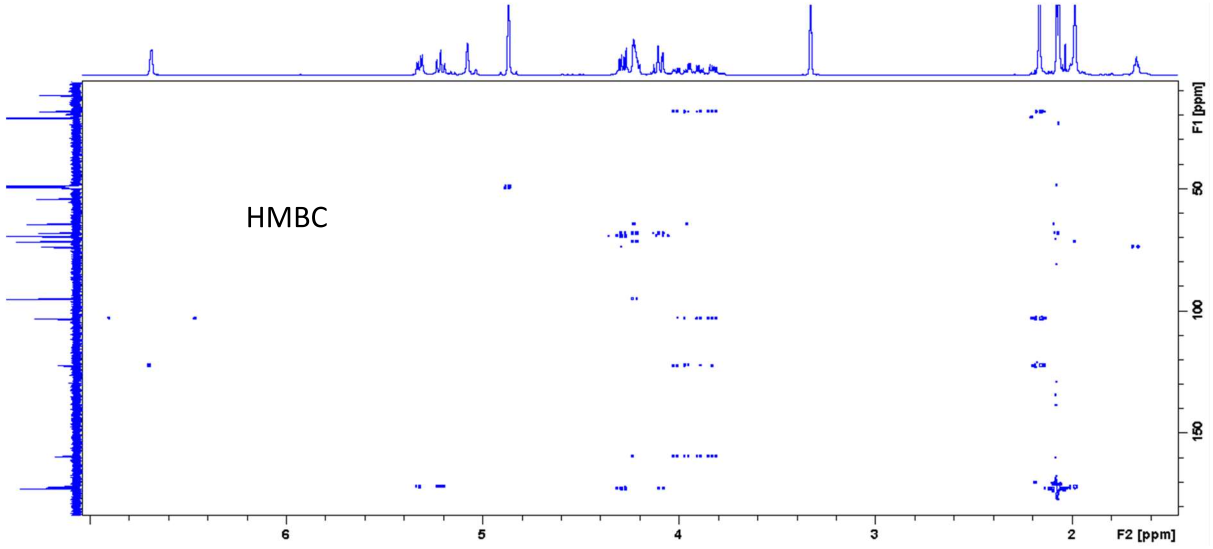

Supplement: Supplementary file 1 — bc2c00169_si_001.pdf [file bc2c00169_si_001.pdf]
